# Electrochemical sensor for simultaneous determination of antiviral favipiravir drug, paracetamol and vitamin C based on host–guest inclusion complex of β-CD/CNTs nanocomposite

**DOI:** 10.1038/s41598-023-45353-3

**Published:** 2023-11-14

**Authors:** Yousef M. Ahmed, Mahmoud A. Eldin, Ahmed Galal, Nada F. Atta

**Affiliations:** Chemistry Department, Faculty of Science, Cairo University, Giza, 12613 Egypt

**Keywords:** Chemistry, Electrochemistry

## Abstract

Favipiravir (FVI) is extensively used as an effective medication against several diverse infectious RNA viruses. It is widely administered as an anti-influenza drug. Combination therapy formed from FVI, paracetamol (PAR) and vitamin C (VC) is needed for treating patients diseased by RNA viruses. Thus, an efficient electrochemical sensor is developed for detecting FVI in human serum samples. The sensor is fabricated by casting a thin layer of carbon nanotubes (CNTs) over a glassy carbon (GC) electrode surface followed by electrodeposition of another layer of β-cyclodextrin (β-CD). Under optimized conditions, the sensor shows excellent catalytic effect for FVI, PAR and VC oxidation in the concentration ranges (0.08 µM → 80 µM), (0.08 µM → 50 µM) and (0.8 µM → 80 µM) with low detection limits of 0.011 μM, 0.042 μM and 0.21 μM, respectively. The combined effect of host–guest interaction ability of β-CD for the drugs, and a large conductive surface area of CNTs improves the sensing performance of the electrode. The sensor exhibits stable response over 4 weeks, good reproducibility, and insignificant interference from common species present in serum samples. The reliability of using the sensor in serum samples shows good recovery of FVI, PAR and VC.

## Introduction

Favipiravir (FVI) is identified as T-705, a nucleobase mimetic. It is first introduced as an inhibitor for infections by influenza virus^1,2^. Recently approved in Japan for the treatment of seasonal influenza as all the currently available anti-influenza drugs (oseltamivir, zanamivir) block the viral neuraminidase ion channels^3^. A wide range of kinds and sub kinds of influenza viruses, including strains resistant to existing anti-influenza drugs are treated by FVI. FVI works through a particular mechanism as it targets the viral RNA polymerases and inhibits RNA replication and directly prevents transcription^4^. Thus, FVI has become a promising candidate drug due to its unique mechanism. Besides, it is widely administered as an anti-influenza drug, FVI is effective against several diverse infectious RNA viruses and acts against other types of epidemic infections, such as COV-19^5–8^. FVI was appropriately used globally as cited in several reports that proved its activity toward severe acute respiratory syndrome coronavirus 2 (SARS-CoV-2)^8–10^, and in patients with severe conditions. SARS-CoV-2 makes patients suffer from permanent threat to their respiratory system and causes mild to moderate symptoms, severe respiratory distress syndrome (ARDS), severe pneumonia, septic shock, and failure of their body organs^11–13^. Further, it caused extraordinary global illness and mortality^14–16^. Fever, sore throat, dry cough, loss of taste and smell, body pain, fatigue, conjunctivitis, nasal congestion, and headache are known symptoms for the severe acute respiratory illness in humans^13,14^. FVI was reported as an effective option for the treatment of patients infected by COV-19, suffering from end stage renal disease (ESRD) and carrying out hemodialysis^17^. Moreover, FVI can work as an antiviral against hepatitis A virus (HAV) infection by introducing nucleotide changes into the HAV genome^18^. Also, FVI shows a broad antiviral activity against filo-, alpha-, bunya-, flavi-, arena- and noro-viruses accompanied with lethal hemorrhagic fever^16,19,20^. Besides, other viruses are life -threatening for humans such as Ebola virus, rabies, Lassa virus and thrombopenia disorder, these viruses can be treated by FVI^2^. Few antiviral drugs are recommended and approved for treating RNA viruses^5^. Based on the unique anti-viral profiles of FVI, determination of FVI is required for routine quality control in pharmaceutical formulations and to assist metabolic and pharmacokinetic studies in human and animal biological samples^21^. Pharmacokinetic studies of FVI indicated that after 2 h of its oral administration the concentration of the drug reaches its maximum in plasma ^22^. In addition, 65% FVI is binding to plasma proteins, and 6.5% is binding to α1-acid^23^. Thus, direct FVI determination is also vital for clinical assays. Thus far, several analytical methods as liquid chromatography-tandem mass spectrometry (LC–MS/MS)^24^, high-performance liquid chromatography (HPLC)^25^, and spectrofluorimetric method^26^ have been reported for the determination of FVI in pharmaceutical or biological samples. The main disadvantages of these methods are time-consuming, multiple pre-sampling steps, less sensitive, high detection limits, limited selectivity, and the instrumentation used in the assays is rather expensive. On the other hand, electrochemical sensors are widely used among the instrumental methods as it provides a suitable approach with high catalytic activity, good sensitivity and chemical stability; cost-effectiveness, time saving, simplicity, and portability^27–29^. So, trace amounts of drugs and other analytes in biological, pharmaceutical, and environmental samples can be determined by electrochemical sensors^30–32^.

Paracetamol is an antipyretic and analgesic drug that is used to temporarily eliminate pain (mild and moderate), and lower high fever. Also, paracetamol blocks the production of definite chemicals known as prostaglandins which are included in pain transmission^31^.

Vitamin C (ascorbic acid) is an antioxidant that the body needs to function and stays healthy. It boosts the immune system and helps prevent cell damage caused by very reactive chemicals (free radicals)^33^. It is a nutrient that the body needs to form and maintains blood vessels, muscle, cartilage, and collagen in bones. Also, it helps fight infections, keeps tissues healthy, heals wounds and protects older people’s cognitive abilities. Other advantages for vitamin C are considered as a medication that helps in treating high blood pressure, reducing chronic and heart diseases, prevents iron deficiency, and decreases uric acid levels in blood (gout attacks)^34^.

Multiwalled carbon nanotubes (CNTs) are made up of concentric cylinders of graphite layers. CNTs are used to improve the thermal stability, hardness, and electrical conductivity of composites. CNTs have been used in sensing applications because of their large effective surface area, and excellent electrochemical properties^35–37^. β-cyclodextrin (β*-*CD) molecule has a cone-shape; it is hydrophobic in the inner surface and hydrophilic in the outer surface due to the existence of hydroxyl groups^38^. The cyclodextrin’s cylindrical shape allows the guest molecule to be kept within the hydrophobic interior while the exterior of the cyclodextrin is hydrophilic and soluble in aqueous phase. CNTs modified with β-CD exhibit high sensitivity for FVI detection because of the good synergism between conducting CNTs and the host guest interaction capabilities of β-cyclodextrins^39^.

In this work, we fabricate a layered sensor by modifying a glassy carbon (GC) electrode surface with two consecutive thin layers of CNTs and β-CD for sensitive determination of FVI in human serum samples and pharmaceutical formulation. Combination therapy formed from FVI, PAR and VC are needed for treating patients diseased by RNA viruses, thus, simultaneous determination of FVI, PAR and VC is studied. The figures of merit for the FVI sensor are examined including sensitivity, detection limit, repeatability, and stability. Recovery tests for FVI, PAR and VC determination in human serum are examined using the modified electrode.

## Experimental

### Chemicals

Multiwalled CNTs (OD/ID × L: 10–15 nm/2–6 nm × 0.1–10 μm, > 90% carbon basis), β-Cyclodextrin (β-CD), ascorbic acid (AA), uric acid (UA), paracetamol (PAR), KH_2_PO_4_, K_2_HPO_4_, H_3_PO_4_, and KOH are purchased from Sigma-Aldrich Chem. Co. (Milwaukee, WI. USA). Favipiravir (FVI) is provided by the National Organization for Drug Control. Supplement Table 1 summarizes the devices used in this work.

### Preparation of tested samples

We examined the applicability of GC/CNT/CD electrode for sensing FVI in samples of real human serum. The serum samples are prepared as mentioned elsewhere^31^. Under optimal experimental conditions we studied the recovery test of FVI in diluted serum sample (diluted five times with PBS) using differential pulse voltammetry (DPV) mode. The specific concentrations of FVI are prepared from 1.0 mM FVI stock solution/0.1 M PBS solution (pH 7.0), standard additions method is used for spiking the FVI drug in diluted serum sample. The same experiment is followed for preparation of PAR and VC specific concentrations and each drug is spiked in diluted serum sample to calculate its recovery percentage.

### Preparation of the FVI sensor

**Figure 1:**
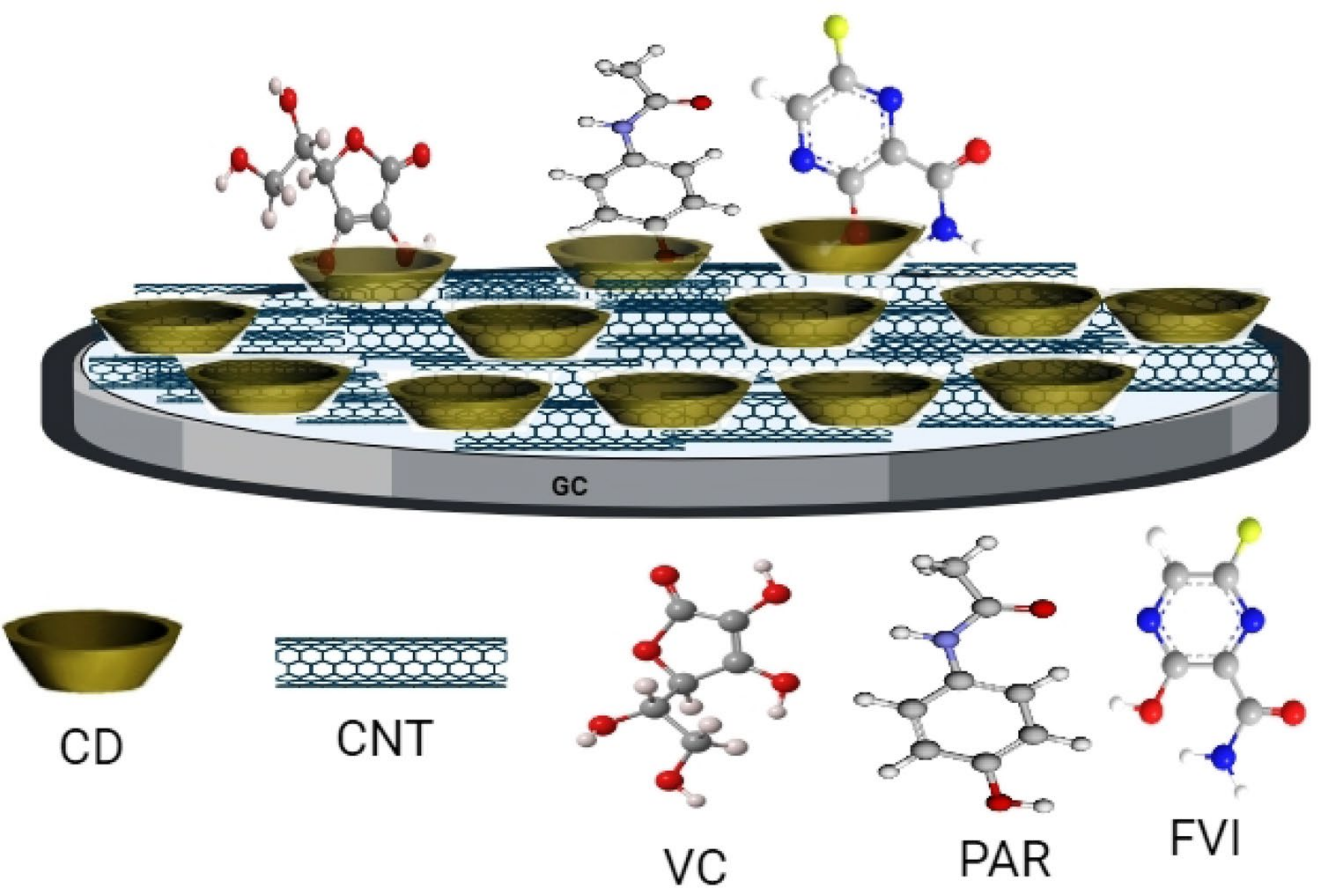
Schematic representation of GC/CNT/CD modified electrode used for the electrochemical oxidation of FVI, PAR and VC.

The GC/CNT/CD sensor (Fig. 1) is prepared as follows: 10 μL from a suspension mixture of (0.5 mg CNTs/1 mL DMF) is cast over the GC surface then the electrode is dried in oven at 50 °C for 10 min. A layer of β-CD is formed electrochemically over a CNTs surface from a solution of 10^–5^ M β-CD/0.1 M PBS (pH 6.0) by cycling the GC/CNT electrode in a potential window from − 2000 to 2500 mV for 3 cycles (optimized). The GC/CNT/CD electrode is left to dry in oven for 10 min at 50 °C.

Electrochemical effective surface areas of the studied electrodes are calculated by running CV experiments in 1.0 mM K_3_[Fe(CN)_6_] system as mentioned elsewhere^40^. The areas for bare GC, GC/CNT and GC/CNT/CD electrodes are found to be 0.0474 cm^2^, 0.1019 cm^2^ and 0.236 cm^2^, respectively, Supplement Fig. 1A–C.

## Results and discussion

### Surface characterization of the modified electrodes

**Figure 2:**
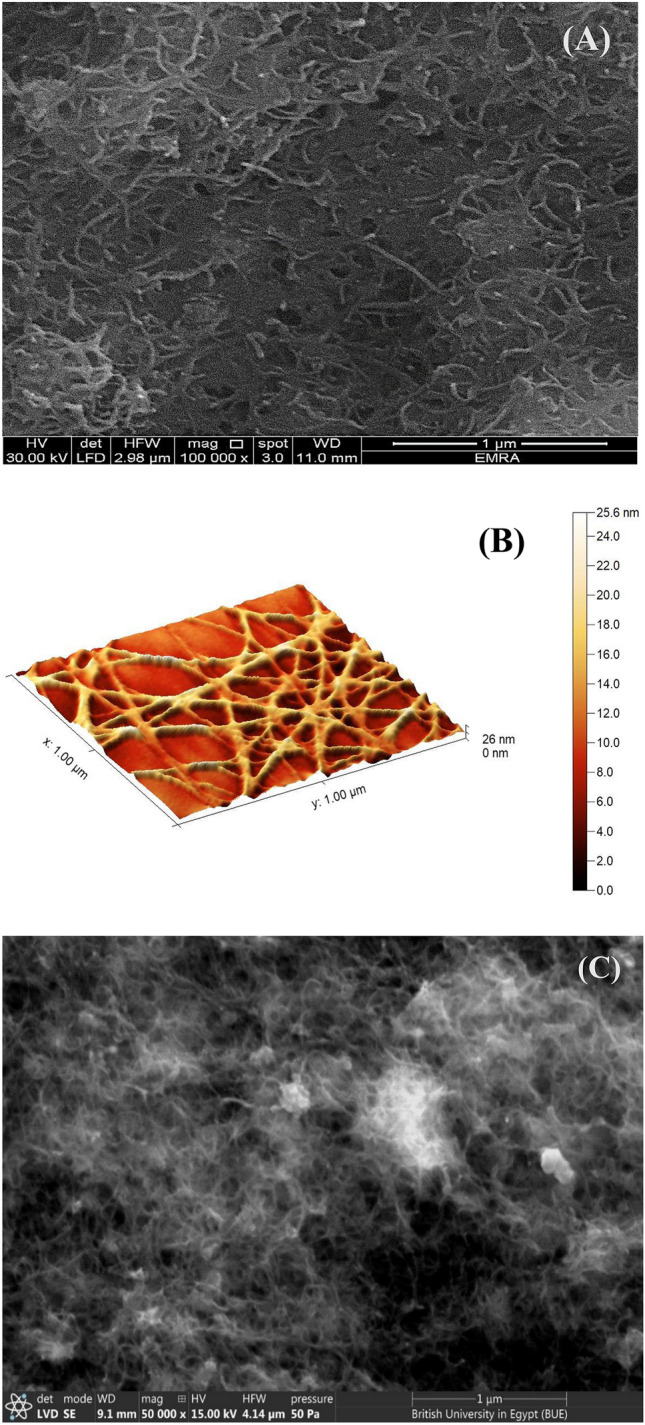

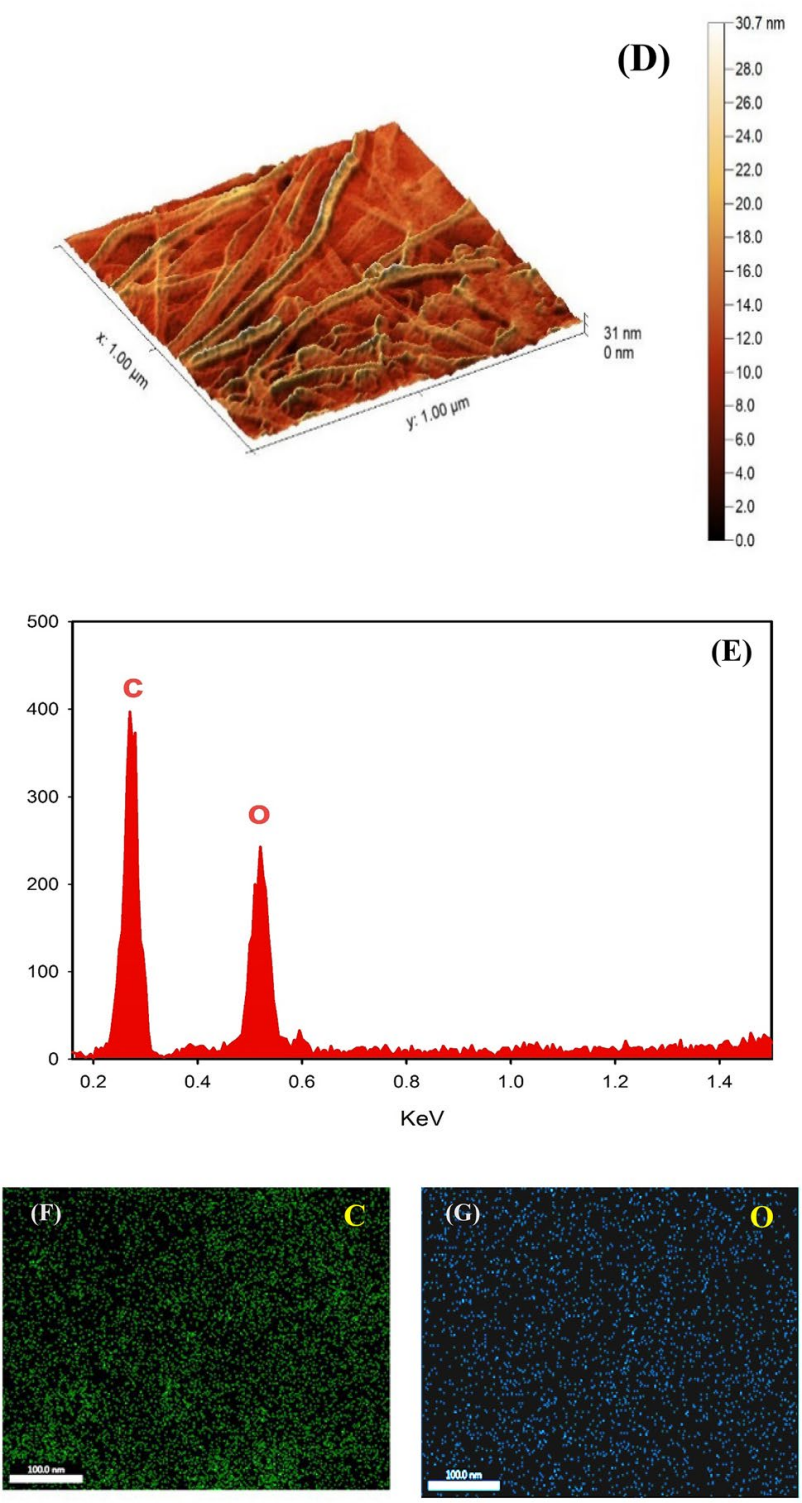
(**A**) SEM of GC/CNT, (**B**) AFM image of GC/CNT, (**C**) SEM of GC/CNT/CD, (**D**) AFM image of GC/CNT/CD, (**E**) EDX of GC/CNT/CD and (**F**,**G**) Elemental mapping of GC/CNT/CD.

Different modified surfaces morphologies are investigated by using scanning electron microscopy (SEM). The atomic force microscope (AFM) is also used to investigate the surface imaging of the film supported on GC electrode. Figure 2A shows the SEM of GC/CNT surface, where a dense network structure of CNTs is observed. AFM illustrates a clear image of CNTs, Fig. 2B. The SEM of GC/CNT/CD shows a blurry vision of interlinked CNTs and few white spots spreading over the surface which may be due to electron charging effects, Fig. 2C. Figure 2D illustrates the AFM image of CNT/CD nanocomposite where undefined CNTs network is appeared after electrodeposition of β-CD. β-CD molecules increase the composite surface area, and the contact areas with the FVI. The combining effect of all composite modifiers: CNTs, and β-CD, affect the electrocatalytic activity of the resulting composite and its conductivity level. Figure 2(E–G) shows EDX and elemental surface mapping of GC/CNT/CD, confirming the presence of all the elements included in the composite. We use FTIR to study the inclusion complexes of the drugs with the β-cyclodextrins. Upon inclusion complexes formation of β-cyclodextrins with FVI, VC, and PAR, the spectra for the inclusion complexes look almost like the pure β-cyclodextrin. The spectrum of each compound and its inclusion complex spectrum are compared as follows: The FTIR spectrum of FVI reveals major peaks at 3412 cm^−1^, 3359 cm^−1^, and 3232 cm^−1^ for antisymmetric and symmetric N–H stretching vibrations and O–H stretching vibration, respectively^41,42^. Other peaks appeared at 1603 cm^−1^, 1659 cm^−1^ and 1265 cm^−1^ for N–H bending vibration, and C = O, C–F stretching vibrations, (Supplement Fig. 2A). The spectrum of β-CD is characterized by an intense peak at 1257 cm^−1^  due to C–O–C stretching vibration and a broad peak at 3435 cm^−1^ assigned to O–H stretching vibration mode, (Supplement Fig. 2B). Upon inclusion complex formation, the peaks of N–H (antisymmetric and symmetric stretching vibrations) and O–H (stretching vibration) at 3412 cm^−1^, 3359 cm^−1^, and 3232 cm^−1^ are not identified, and are disappeared under the broad peak of O–H at 3436 cm^−1^, also the C–F stretching vibration at 1265 cm^−1^ is not recognized as shown in FTIR inclusion spectrum (Supplement Fig. 2C). This suggests the formation of inclusion complex because of the good interaction between the β-CD host cavity and the FVI. Also, the FTIR spectra of the inclusion complexes of β-cyclodextrins with PAR and VC are compared with the FTIR spectra of PAR and VC, (Supplement Fig. 2D–G). Also, upon inclusion complex formation of β-CD with PAR, some peaks in PAR spectrum are not identified in its inclusion spectrum such as peaks at 3162 cm^−1^ (–OH stretching), 3325 cm^−1^ (N–H amide stretching), 1650 cm^−1^ (C = O stretching), and 1612 cm^−1^ (C = C stretching), or their intensities diminished such as 1563 cm^−1^ (N–H amide II bending), 1508 cm^−1^ (asymmetrical C–H bending), and 1432 cm^−1^ (C–C stretching)^43,44^, Supplement Fig. 2E. In inclusion complex spectrum of β-CD with VC, the stretching vibrations of enol-hydroxyl, and carbonyl (C = O) at 1613 cm^1^ and 1755 cm^−1^, respectively in the VC spectrum are not identified in its inclusion spectrum. Also, many peaks in the VC spectrum disappeared after complexation such as (= C–H stretching) at 3034 cm^−1^ and (the four O–H stretching) at 3527 cm^−1^, 3412 cm^−1^, 3317 cm^−1^, and 3220 cm^−1^, they are hidden in the broad stretching peak of OH at 3403 cm^−1^ as shown in its inclusion spectrum^45^, Supplement Fig. 2G. These results suggest that the PAR and VC are inserted within the β-CD cavities with a good interaction between them and the cyclodextrins^43–45^.

Supplement Fig. 2H shows the Raman spectrum of CNT/CD composite, confirming the presence of all the constituents included in the composite.

### Electrochemical impedance spectroscopy “EIS”

**Figure 3:**
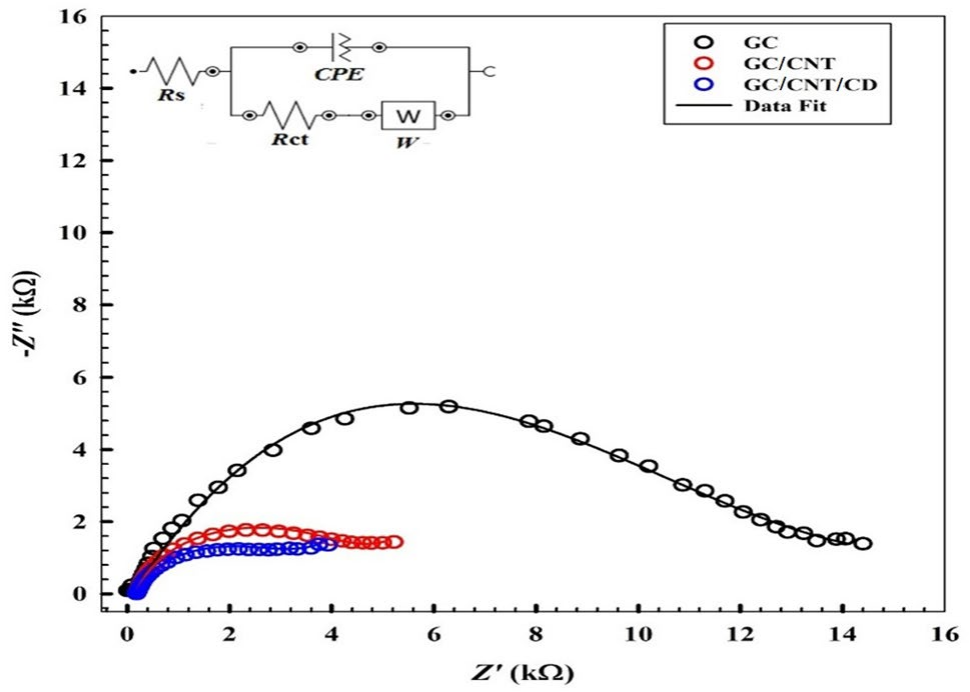
Typical impedance spectra presented in the form of the Nyquist plots for GC, GC/CNT, GC/CNT/CD in 1.0 mM of FVI/0.1 M PBS/pH 7.0 (Symbols and solid lines represent the experimental measurements and the computer fitting of impedance spectra, respectively). Inset: Equivalent circuit used in the fit procedure of the impedance spectra.

Electrochemical impedance spectroscopy is an effective technique to investigate the interface properties of modified electrodes. A suggested illustration of the surface modification and its corresponding variation in the charge transfer kinetics and capacitive components of the system can be shown by the aid of EIS. The impedance spectra in the form of Nyquist plots for GC, GC/CNT, and GC/CNT/CD electrodes in FVI are shown in Fig. 3. EIS experiments are applied in 1.0 mM FVI/0.1 M PBS/pH 7.0 at an AC frequency in the range of 0.1 Hz to 100 kHz at the corresponding oxidation potentials of FVI electrodes. Upon modification, the charge transfer resistance decreased reflecting the enhancement of the charge transfer rate owing to the interactive impact of the individual components of the composite surface. A modified Randle's circuit is used to fit the corresponding EIS data of FVI (Inset of Fig. 3). The fitting data using this circuit are represented as solid lines in EIS spectra and a good agreement was obtained between the experimental and fitting data. So, the equivalent circuit contained: Rs (ohmic solution resistance), Rct (charge transfer resistance which is related to the kinetics of the charge transfer reaction at the interface.), Y^o^ (the capacitance of the interfacial double layer which is represented by a constant phase element with its corresponding component n), and W (Warburg admittance due to the diffusion of FVI analyte from solution bulk to electrode surface). Table 1 lists the fitting data for FVI corresponding to data in Fig. 3. A semicircle with large diameter is obtained due to high electron transfer resistance at GC electrode as shown in Fig. 3. At GC/CNT, a semicircle with small diameter is obtained, reflecting a lower charge transfer resistance compared to GC electrode. But only semi-linear part is depicted at GC/CNT/CD electrode reflecting a lower charge transfer resistance and faster charge transfer kinetics, Fig. 3. There is a decrease in the value of Rct of GC/CNT/CD upon modification compared to GC, and GC/CNT, indicating the characteristic features of the proposed surface. In addition, a noticeable increase in Y^o^ value of GC/CNT/CD compared to GC, and GC/CNT with n < 1, confirming the capacitive nature of the proposed composite.

**Table 1:** EIS fitting data corresponding to results in Fig. 3.

	Rs (Ω)	Rct (kΩ)	CPE Y° (μMho.s^n^) n	W Y° (μMho.s^1/2^)	χ^2^
GC	110	14.3	7.12 × 10^−6^	8.58 × 10^−4^	0.391
			0.771		
GC/CNT	185	4.16	3.10 × 10^−5^	8.74 × 10^−4^	0.127
			0.870		
GC/CNT/CD	184	2.96	5.30 × 10^−5^	8.89 × 10^−4^	0.126
			0.840		

### Electrochemistry of the modified electrodes

**Figure 4:**
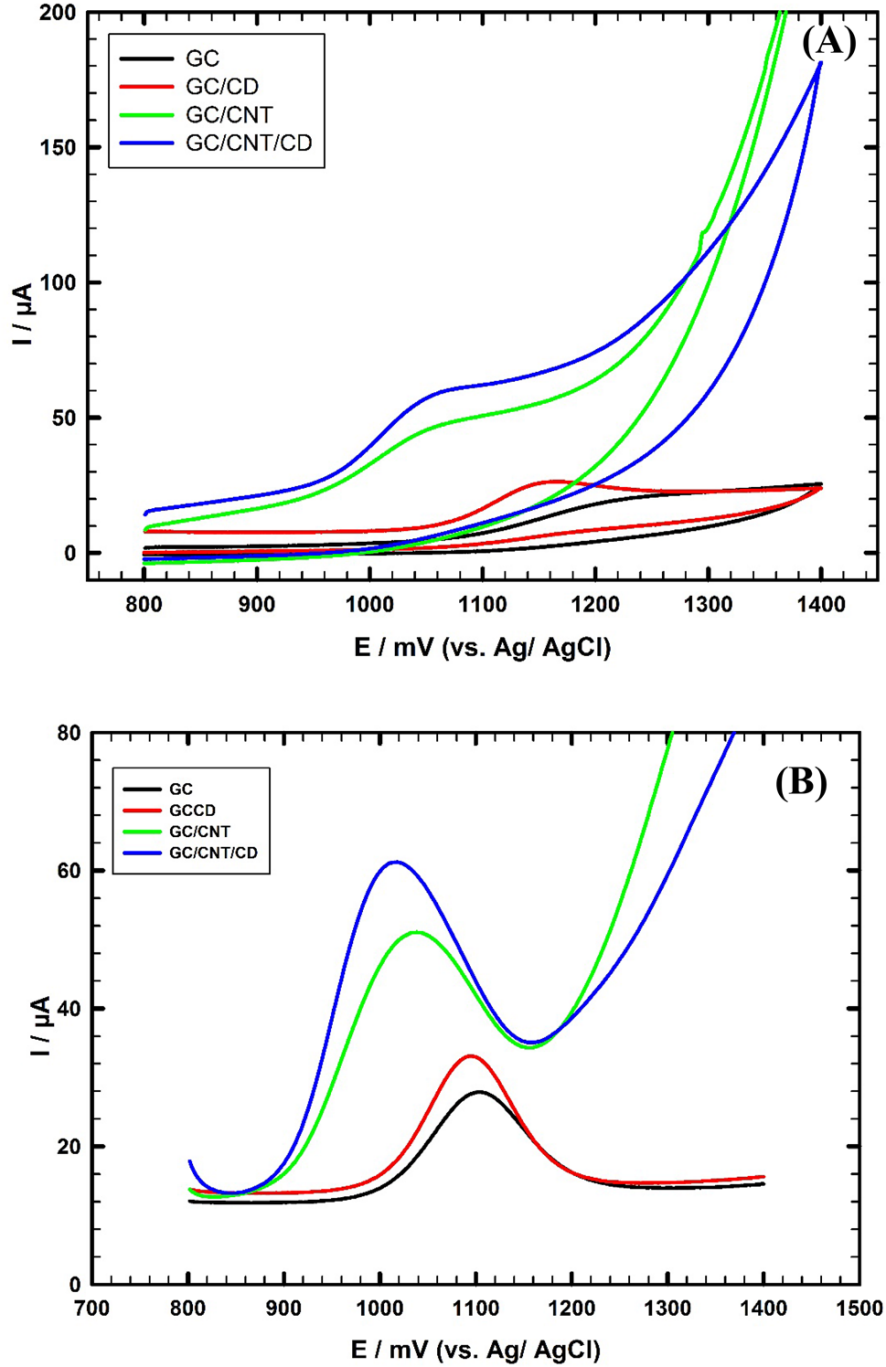
(**A**) DPVs of 1.0 mM FVI/0.1 M PBS (pH 7.0), scan rate 50 mV/s using different working electrodes. (**B**) DPVs of 1.0 mM FVI/0.1 M PBS (pH 7.0) using different working electrodes.

The catalytic activity of the different working electrodes toward the electrochemical oxidation of 1.0 mM FVI/0.1M PBS/pH 7.0 is studied. Cyclic voltammetry (CV) is performed to compare the electrochemical sensing performance of different working electrodes, Fig. 4A. The sensitivity of bare GC and GC/CD electrodes toward the electrochemical oxidation of FVI is low where the oxidation current values are 10.1 µA, and 16.6 µA at potential values 1236 mV and 1146 mV, respectively. The modified electrodes GC/CNT and GC/CNT/CD show oxidation peaks, but no reduction peaks, illustrating that the electrochemical oxidation of FVI is an irreversible process. Two electrons and one proton are involved in the FVI oxidation. The GC/CNT electrode shows higher oxidation current response of 25.5 µA at 1052 mV. Further modification of the GC/CNT electrode surface with β-CD increases the oxidation current response up to 35.3 μA at 1041 mV. The oxidation peak potential of FVI shifts to a less positive value i.e., allows thermodynamically favored oxidation reaction compared to GC and GC/CNT electrodes. The obtained results are due to the combined effect of host–guest interaction ability of β-CD with the FVI, and a large conductive surface area of CNTs which results in significant enhancement of the composite sensitivity. This is due to the good synergism between CNTs and β-CD. β-CD accumulates the FVI due to the hydrophobicity of the inner core, and the hydrophilic outer core facilitates more oxidation of FVI. This increases the catalytic effect of the proposed composite by increasing the rate of charge exchange at the interface.

We ran the experiment by another voltammetry mode namely DPV to illustrate the difference between the electrochemical behavior of the modified electrodes toward FVI. DPV is a sensitive mode and useful for detecting and analyzing small changes in the electrochemical behavior of a studied system. Thus, the electrochemical response of FVI is studied at GC/CD, GC/CNT and GC/CNT/CD electrodes using DPV, (Fig. 4B). Compared to the GC electrode, the oxidation peak currents (I_pa_) of FVI significantly increased at the GC/CD, GC/CNT, and GC/CNT/CD electrodes by 1.3, 2.5, and 3.1 folds, respectively and the oxidation peak potentials (Ep_a_) shifted to less positive values by 14 mV, 66 mV, and 90 mV, respectively. The large surface area of CNTs available for the reaction is more suitable for the assembly of cyclodextrins. Cyclodextrins have unique cavities; these unique structures enable good binding with various guest compounds into their cavities forming stable host–guest inclusion complexes or nanostructure supramolecular complexes. The suggested mechanisms are as follows: the FVI ring is inserted in the β-CD cavity, the β-CD structure contains large number of OH groups. Hydrogen bond (weak non-covalent interaction) is formed between the O–H group of β-CD and the O–H group of FVI. Also, β-CD can form inclusion complexes with PAR and VC. The PAR ring is inserted in the β-CD cavity with the OH group directed toward the CD-cavity and a good interaction occurs between the β-CD and the hydroxyl group of PAR. The VC ring is inserted in the β-CD cavity with the two OH groups directed toward the CD-cavity and hydrogen bonds are formed between the β-CD and the two O–H groups of VC. As a result, these hydrogen bonds activate and decrease the bonding energy of the hydroxyl groups of drugs, facilitate the electrons transfer reactions, which results in enhancing the rate of electron transfer processes^46–49^.

### Effect of scan rate

CV is used to study the effect of applying different scan rates (10–100 mV s^–1^) on the oxidation peak current of 1.0 mM FVI/0.1 M PBS/pH 7.0 using the optimized sensor as shown in Supplement Fig. 3. Also, scan rate is studied to examine the number of electrons transferred during the FVI oxidation process. The FVI oxidation current increases with increasing the scan rate. Inset A of Supplement Fig. 3 shows the linear regression relationship of peak oxidation current (I_p_) versus square root of scan rate and can be expressed as follows:

$${\text{I}}_{{\text{p}}} \left( {\mu {\text{A}}} \right)\, = \,{4}.{13}\, + \,{339}.{1}v^{{0.{5}}} \left( {{\text{V}}.{\text{s}}^{{ - {1}}} } \right)^{{0.{5}}} ({\text{R}}^{{2}} \, = \,0.{993}).$$

The linear relationship of I_p_ versus *v*^½^ indicates that the FVI oxidation is a diffusion-controlled process^50,51^.

There is another evidence of the diffusion-controlled process by plotting a relation between log I and log *v* as shown in inset B of Supplement Fig. 3. Linear relationship is obtained between log I_p_ versus log *v* and can be expressed as follows:

$${\text{Log I}}_{{\text{p}}} \left( {\mu {\text{A}}} \right)\, = \,{2}.{51} - 0.{\text{471Log}}v\left( {{\text{V}}.{\text{s}}^{{ - {1}}} } \right) \, ({\text{R}}^{{2}} \, = \,0.{997}).$$

The slope of this relation is 0.471 which is close to the theoretical value of 0.50 for a diffusion-controlled process^52^.

Inset C of supplement Fig. 3 shows the linear relationship between the peak potential of FVI versus the logarithm of the scan rate and can be expressed as follows:

$${\text{E}}_{{\text{p}}} \left( {\text{V}} \right)\, = \,{24}.{5}\, - \,0.0{\text{41 Log}}v\left( {{\text{Vs}}^{{ - {1}}} } \right) ({\text{R}}^{{2}} \, = \,0.{984}).$$

The inset C illustrates that as the scanning speed increases, the peak potential decreases, indicating that the electrochemical reaction is a typical irreversible process^53^.

For irreversible electrochemical reactions, E_p_ can be obtained by the following formula^53^.

$${\text{E}}_{{\text{p}}} \left( {\text{V}} \right)\, = \,\left( { - \,{2}.{\text{3RT}}/\alpha {\text{nF}}} \right){\text{ log}}v\left( {{\text{V}}/{\text{s}}} \right)\, + \,{\text{Constant}},$$

where n is the total number of electrons transferred, α is the electron transfer coefficient, R = 8.314 J/K mol, F = 96,480 C/mol, T = 298 K. α can be calculated using the following formula^54^.

$$\alpha \, = \,{47}.{7}/\left( {{\text{E}}_{{\text{p}}} \, - \,{\text{E}}_{{{\text{p}}/{2}}} } \right),$$

where E_p/2_ is the potential where the current is at half the peak value. The calculated value of α is 0.733. From the slope of the linear relation of E_p_ versus log *v*, the value of n can be calculated, it is approximately equal to 1.98 = 2, indicating that two electrons are involved in the electrochemical oxidation of FVI.

### Effect of pH

**Figure 5:**
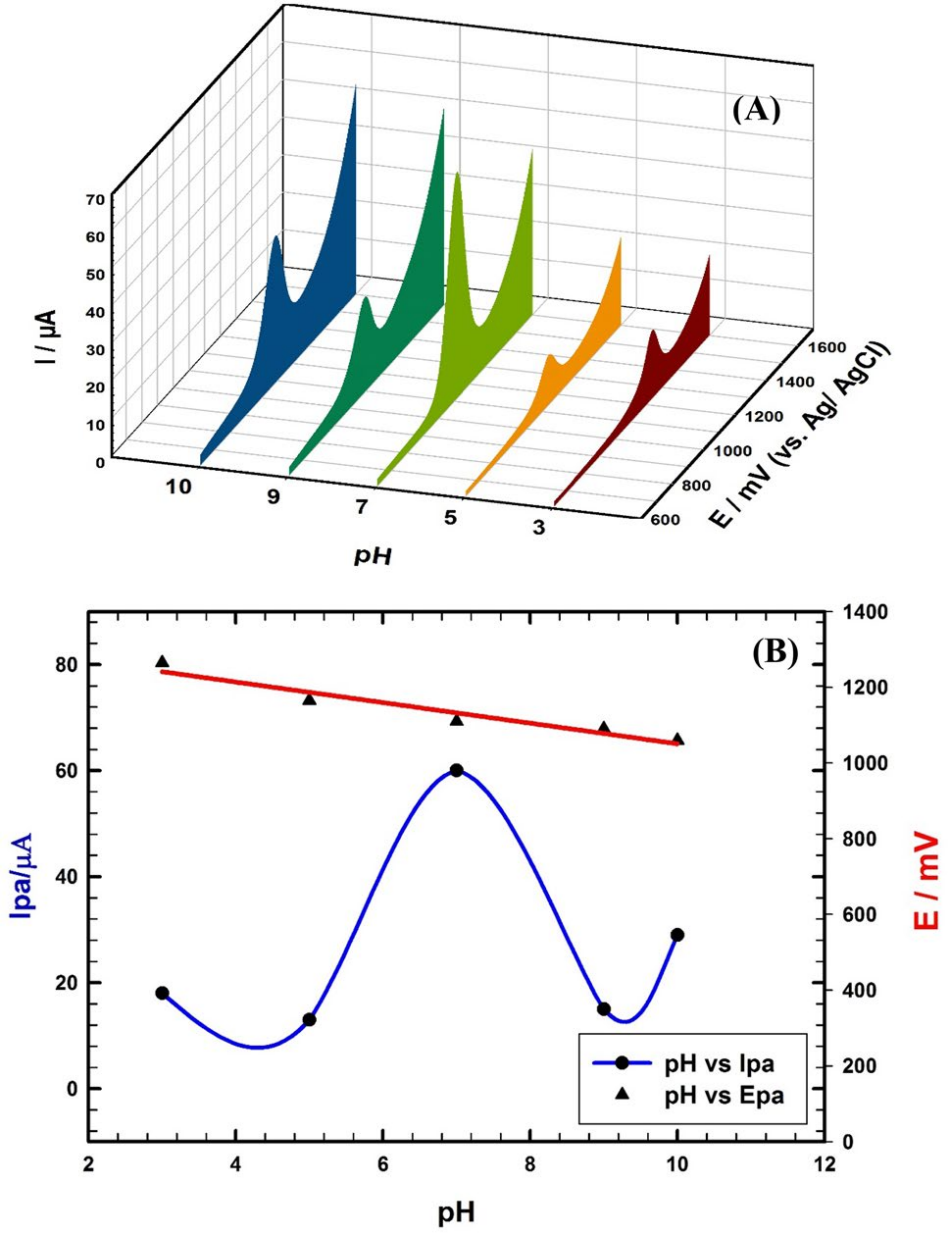
(**A**) CVs of 1.0 mM FVI/0.1 M PBS with different pH values, scan rate 50 mV/s using GC/CNT/CD electrode. (**B**) Relation between oxidation peak potential and pH; and relation between oxidation peak current and pH.

The pH of buffer solution has an important effect in both the oxidation potential and the oxidation current of FVI. Differential cyclic voltammetry is used to study the pH-dependent behavior of 1.0 mM FVI oxidation as shown in Fig. 5A. Figure 5B represents the variation of both peak oxidation current (I_p_) and peak oxidation potential (E_p_) as a function of electrolyte pH. I_p_ increases with pH up to 7.0, then there is a sharp decrease as shown in Fig. 5B. At pH 7.0 the electrode's sensitivity and selectivity for detection FVI reach maximum value^55,56^. At pH > 7.0, the FVI oxidation peak current decreases due to the hydrolysis of FVI and its adsorption decreases on the electrode surface^54^. The relationship between oxidation peak potential and the pH is presented in Fig. 5B. The oxidation peak potential decreases with an increase in pH, which indicates that protons are included in the oxidation process. The relation can be expressed by the following linear regression equation:

$${\text{E }}\left( {{\text{mV}}} \right)\, = \,{1346} - {29}.{\text{8 pH\,\, R}}^{{2}} \, = \,0.{997}.$$

The proton/electron ratio (m/n) was found to be 1:2 using the Nernst equation (dE_p_/dpH = 0.059 m/n)^54^. Thus, oxidation of FVI at the modified sensor involves 2 electrons and 1 proton. In addition, pH study is performed for PAR and VC and the highest peak current responses are obtained at pH 7.0 and 3.0, respectively as shown in Supplement Fig. 4(A,B). We chose the PBS of pH 7.0 which is nearly the same as pH of biological fluids to run all experiments.

### Reproducibility and stability

The current response of 1.0 mM FVI/0.1 M PBS/pH 7.0 at three different electrodes prepared independently under similar construction conditions is investigated to determine the fabrication reproducibility of the sensor. Acceptable fabrication reproducibility is obtained as the value of RSD is 2.32%. The intra-day and inter-day reproducibility are checked by evaluating the performance of the same sensor for four runs in 0.1 mM FVI/0.1 M PBS or using four sensors made independently and testing them in 0.1 mM FVI/0.1 M PBS in four separate measurements, respectively. The calculated RSD values are 1.58%, and 1.86% for intra-day and inter-day precisions, respectively. These low values reflect the applicability of the proposed sensor towards FVI determination with stable response and good precision, Supplement Fig. 5A.

CV is used to examine the current response stability of the sensor toward FVI oxidation. The sensor shows good stability after 25 runs in 1.0 mM FVI as shown in Supplement Fig. 5B, and for long term stability of 4 weeks, the electrochemical response of the electrode maintains 95.8% of its initial current value, Supplement Fig. 5C. Also, long term stability of 10 days is examined for VC and PAR and the oxidation current responses maintain 98.3% and 97.6%, respectively of their initial current values, Supplement Fig.5D. Thus, the current response stability of the sensor is attributed to the composite materials stability.

### Robustness

The robustness of this method is evaluated upon the impact of minor alterations in the experimental conditions. The studied parameters are time before running the experiment (2 min ± 20 s) and pH change (7.0 ± 0.2). The relative standard deviations are 1.33% and 2.46%, respectively confirm the steadiness of the current response stability.

### Voltammetry determination of FVI in real sample

**Figure 6:**
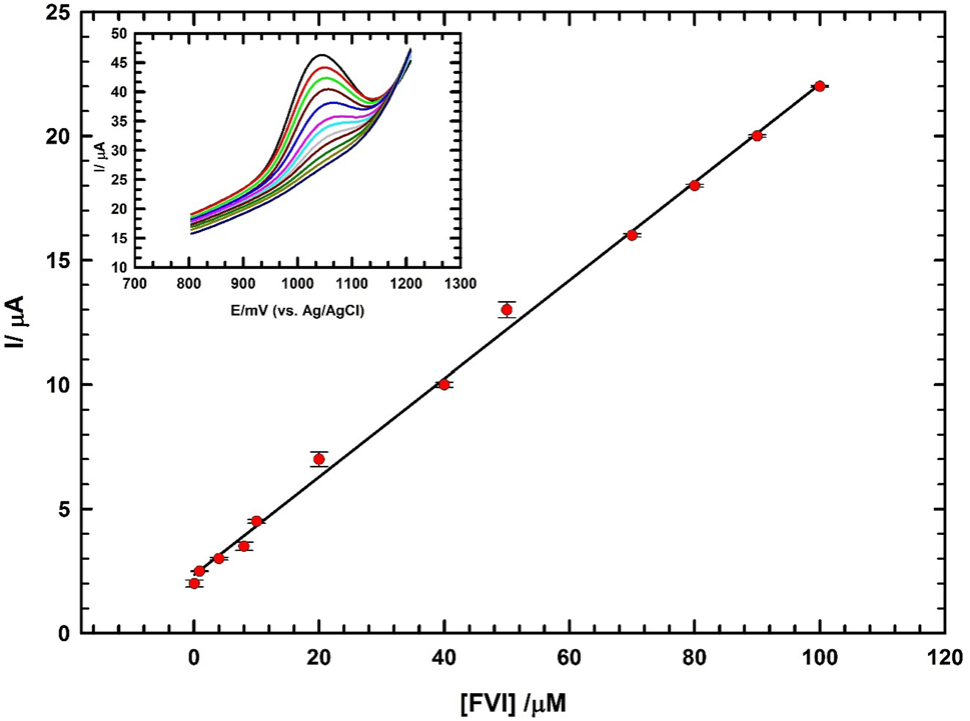
Calibration of FVI in the concentration range (0.07 µM to 100 µM) in dilute human serum/0.1 M PBS (pH 7.0); inset the corresponding DPVs using GC/CNT/CD electrode.

In this section we apply DPV mode for the determination of FVI in human serum sample/0.1 M PBS/pH 7.0 using the GC/CNT/CD electrode. After increasing the concentration of FVI in diluted serum sample, typical voltammograms are obtained with the increase of FVI concentration in the range (0.07 µM → 100 µM) as shown in (Fig. 6; inset). According to ICH guidelines the proposed method is validated with respect to linearity, detection limit (DL), and quantification limit (QL)^57^. Figure 6 indicates the obtained linear range covered the FVI concentration from (0.07 µM → 100 µM). The corresponding regression equation is:

$${\text{I}}_{{\text{p}}} \left( {\mu {\text{A}}} \right)\, = \,\left( {0.{197}\, \pm \,0.00{435}} \right){\text{ C }}\left( {\mu {\text{M}}} \right)\, + \,\left( {{2}.{34}\, \pm \,0.0{581}} \right), \, ({\text{R}}^{{2}} \, = \,0.{998},{\text{ n}}\, = \,{3}).$$

The DL and QL are 1.06 nM and 3.52 nM, respectively. DL and QL are determined according to (S/N = 3)^57^. The performance of the sensor for FVI detection is compared with other cited modified sensors^21,54,58–61^. The sensor offers reasonable linear range for FVI detection with DL lower than other used modified electrodes as illustrated in Supplement Table 2. Thus, the sensor can be applied with acceptable confidence for the determination of FVI levels in serum samples.

### Simultaneous determination of FVI, PAR and VC

Combination therapy formed from FVI, PAR and VC is needed for treating patients diseased by RNA viruses. FVI and PAR can be used together as part of a treatment regimen for viral infections. FVI targets the viral RNA polymerases and inhibits RNA replication and directly prevents transcription. PAR is a pain reliever and fever reducer that can help manage symptoms associated with viral infections. Vitamin C is an antioxidant the body needs to function and stays healthy. It helps fight infections, boosts the immune system, prevents cell damage caused by very reactive chemicals and keeps tissues healthy. It is important to use these drugs appropriately and according to the recommended dose and duration to avoid adverse effects.

**Figure 7:**
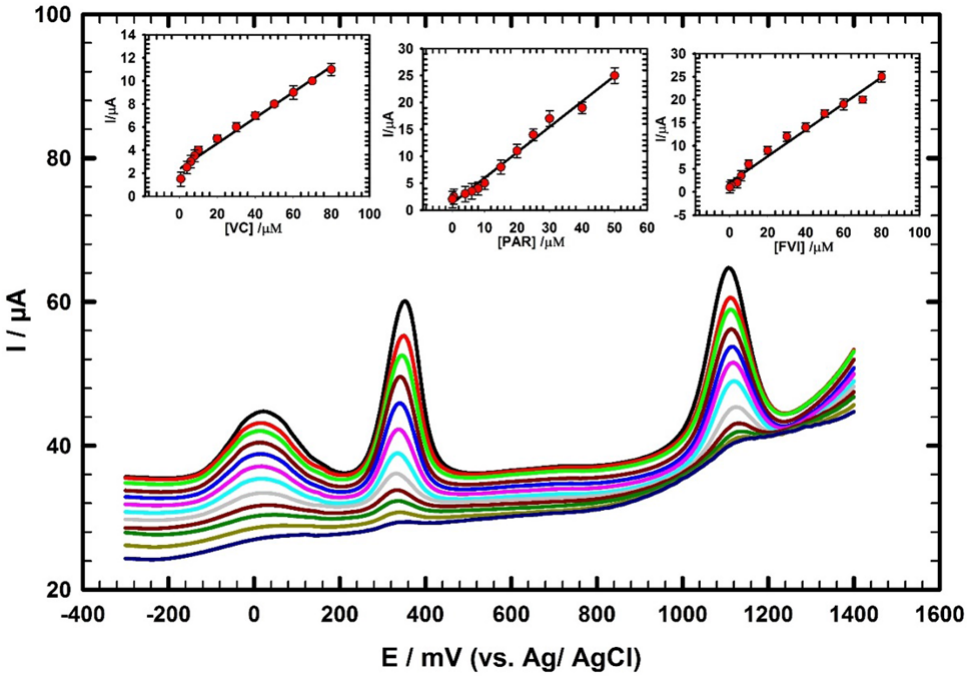
DPVs of simultaneous determination of FVI, PAR and VC in concentration ranges (0.08 → 80 µM), (0.08 → 50 µM), and (0.8 → 80 µM) in dilute human serum/0.1 M PBS pH 7.0; inset: the corresponding calibration curves for FVI, PAR and VC using GC/CNT/CD electrode.

Thus, it is important to examine the simultaneous determination of FVI, PAR and VC in dilute human serum sample employing the GC/CNT/CD modified electrode. Highly resolved oxidation signals of FVI, PAR and VC with large potential separation between their oxidation peaks are obtained as shown in Fig. 7. The oxidation current responses of the FVI, PAR and VC compounds increased linearly with the increase of their concentrations in the ranges (0.08 → 80 µM), (0.08 → 50 µM), and (0.8 → 80 µM), respectively as shown in (Fig. 7; insets). The linear regression equations are as follows:

$${\text{I}}_{{\text{p}}} \left( {\mu {\text{A}}} \right)\, = \,\left( {0.{285}\, \pm \,0.00{23}} \right){\text{ C}}_{{{\text{FVI}}}} \left( {\mu {\text{M}}} \right)\, + \,\left( {{2}.0{2}\, \pm \,0.{152}} \right), \, ({\text{R}}^{{2}} \, = \,0.{99}0,{\text{ n}}\, = \,{3}).$$

$${\text{I}}_{{\text{p}}} \left( {\mu {\text{A}}} \right)\, = \,\left( {0.{473}\, \pm \,0.00{43}} \right){\text{ C}}_{{{\text{PAR}}}} \left( {\mu {\text{M}}} \right)\, + \,\left( {{1}.{27}\, \pm \,0.{194}} \right), \, ({\text{R}}^{{2}} \, = \,0.{995},{\text{ n}}\, = \,{3}).$$

$${\text{I}}_{{\text{p}}} \left( {\mu {\text{A}}} \right)\, = \,\left( {0.{113}\, \pm \,0.00{28}} \right){\text{ C}}_{{{\text{VC}}}} \left( {\mu {\text{M}}} \right)\, + \,\left( {{2}.{67}\, \pm \,0.{1}0{5}} \right), \, ({\text{R}}^{{2}} \, = \,0.{99}0,{\text{ n}}\, = \,{3}).$$

The DLs are 0.011 μM, 0.042 μM and 0.21 μM, respectively.

Therefore, an electrochemical sensor can be a useful tool in developing an effective treatment for patients with viral infections.

### Simultaneous determination of FVI and PAR

**Figure 8:**
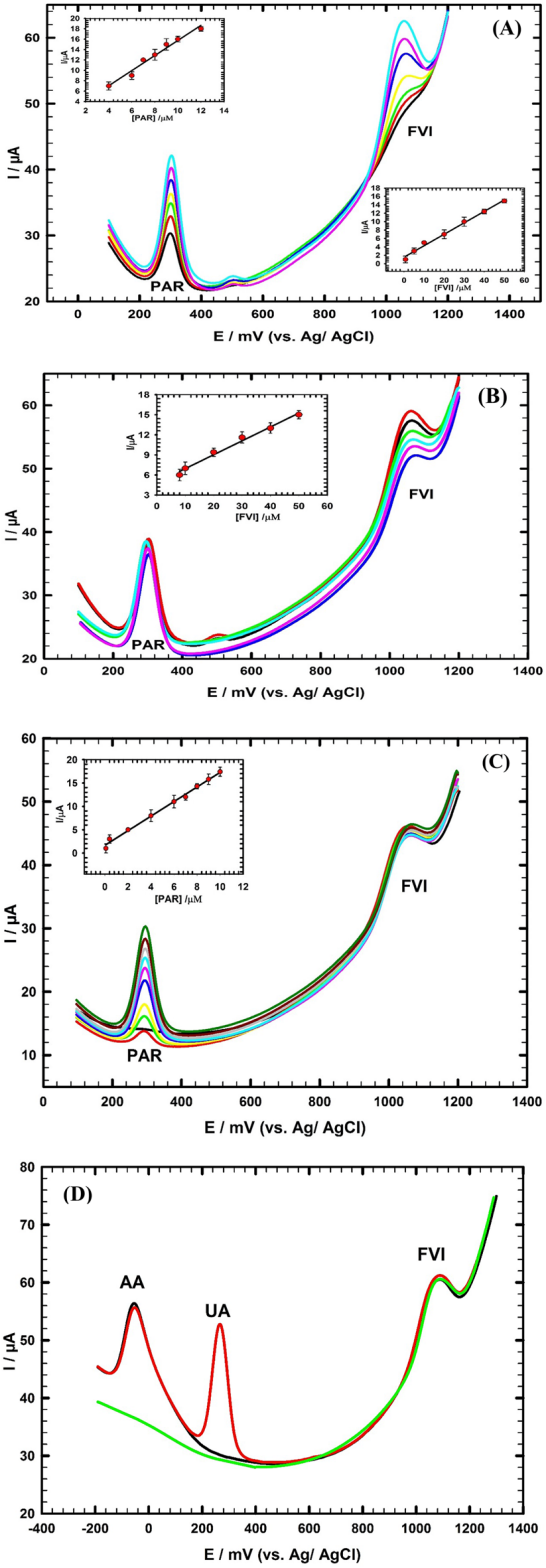
(**A**) DPVs of simultaneous determination of FVI and PAR in concentration ranges (0.7 → 50 µM) and (4 → 12 µM)/0.1 M PBS pH 7.0; inset: the corresponding calibration curves for PAR and FVI. (**B**) DPVs of FVI in the concentration range from (8 μM to 50 μM) in presence of constant concentration 8 μM of PAR in 0.1 M PBS/pH 7.0; inset: the corresponding calibration curve for FVI. (**C**) DPVs of PAR in the concentration range from (0.08 μM to 10 μM) in presence of constant concentration 20 μM of FVI in 0.1 M PBS/pH 7.0; inset: the corresponding calibration curve for PAR. (**D**) DPVs of Ternary mixture containing 800 µM AA, 30 µM FVI in presence of 80 µM UA (red), in absence of UA (black), and in absence of AA and UA (green) at GC/CNT/CD in 0.1 M PBS/pH 7.0.

PAR can help improving a patient's comfort level by reducing fever and relieving pain, while FVI is an antiviral medication that works by inhibiting the replication of the virus in the body. Thus, FVI and PAR can be used together to treat viral infections and their symptoms. Therefore, it is essential to study the simultaneous determination of FVI and PAR in their mixture using the GC/CNT/CD electrode. This study is performed to evaluate the possible mutual interference for separation of a mixture of FVI and PAR. Figure 8A illustrates the highly resolved oxidation peaks of FVI and PAR with significant potential separation between their oxidation signals. The oxidation current responses of FVI and PAR increased linearly with the increase of their concentrations in the ranges (0.7 → 50 µM) and (4 → 12 µM), respectively as shown in (Fig. 8A; insets). The regression equations for the linear relations of PAR and FVI are:

$${\text{I}}_{{\text{p}}} \left( {\mu {\text{A}}} \right)\, = \,\left( {0.{272}\, \pm \,0.00{14}} \right){\text{ C}}_{{{\text{FVI}}}} \left( {\mu {\text{M}}} \right)\, + \,\left( {{1}.{56}\, \pm \,0.{127}} \right), \, ({\text{R}}^{{2}} \, = \,0.{995},{\text{ n}}\, = \,{3}).$$

$${\text{I}}_{{\text{p}}} \left( {\mu {\text{A}}} \right)\, = \,\left( {0.{452}\, \pm \,0.00{21}} \right){\text{ C}}_{{{\text{PAR}}}} \left( {\mu {\text{M}}} \right)\, + \,\left( {{1}.{24}\, \pm \,0.{112}} \right), \, ({\text{R}}^{{2}} \, = \,0.{997},{\text{ n}}\, = \,{3}).$$

Accurate determination of the level of FVI without interference from PAR in patient's biological fluids can help patients to monitor the appropriate dose and avoid potential side effects or adverse reactions. Figure 8B shows the DPV pattern where the concentration of PAR is remained constant at (8 μM) and the FVI concentration is changed in the range (8 µM → 50 µM). The FVI peak current response increased linearly with the increase of its concentration in the range (8 µM → 50 µM) as shown in (Fig. 8B; inset). The regression equation for the linear relation of FVI is:

$${\text{I}}_{{\text{p}}} \left( {\mu {\text{A}}} \right)\, = \,\left( {0.{2}0{8}\, \pm \,0.00{67}} \right){\text{ C}}_{{{\text{FVI}}}} \left( {\mu {\text{M}}} \right)\, + \,\left( {{4}.{84}\, \pm \,0.{127}} \right), \, ({\text{R}}^{{2}} \, = \,0.{992},{\text{ n}}\, = \,{3}).$$

Figure 8C shows the DPV pattern where the PAR concentration is changed in the range (0.08 µM → 10 µM) and the concentration of FVI is remained constant at (20 μM). The PAR peak current response increased linearly with the increase of its concentration in the range (0.08 µM → 10 µM) as shown in (Fig. 8C; inset). The regression equation for the linear relation of PAR is as follows:

$${\text{I}}_{{\text{p}}} \left( {\mu {\text{A}}} \right)\, = \,\left( {0.{56}\, \pm \,0.00{23}} \right){\text{ C}}_{{{\text{PAR}}}} \left( {\mu {\text{M}}} \right)\, + \,\left( {{1}.{67}\, \pm \,0.{1}0{9}} \right), \, ({\text{R}}^{{2}} \, = \,0.{995},{\text{ n}}\, = \,{3}).$$

### Interference study

It is necessary to examine the ability of the proposed sensor to selectively and simultaneously discriminate between the studied drug and the interfering species present in the human fluids. AA and UA are two common interfering compounds that can be found in human body fluids. AA and UA can interfere with the accurate detection of the studied drug. Therefore, it is important to consider and address their potential influence when developing a proposed method for detecting FVI drug in biological samples.

Figure 8D indicates the DPV pattern of a ternary mixture containing AA (800 μM), UA (80 μM), and FVI (30 μM). The mixture is analyzed at GC/CNT/CD modified electrode. Three separated and clearly defined oxidation peaks are observed in the ternary mixture. This demonstrates that the GC/CNT/CD electrode is effective in separating and detecting the individual components of the ternary mixture. Also, we examined the sensor in absence of UA, and in absence of AA and UA, the same current responses of FVI are obtained in the two cases, indicating the stability of the FVI current response and the anti-interference ability of the sensor.

### Recovery

Recovery experiments are carried out by standard additions of known concentrations of FVI, PAR, and VC into diluted human serum sample to study the accuracy of the proposed method. By comparing the calculated concentrations of the drugs from their calibration curves with the added concentrations in the sample, Supplement Fig. 6, the recovery percentages are determined. The analysis is repeated three times after each addition of known concentrations of FVI, PAR and VC in the sample and the average current value is calculated for every drug. Table 2 lists the obtained results. The recovery and RSD values for FVI, PAR and VC are in the ranges of (99.04–102.7%) and (0.72–1.61%); (99.05–101.12%) and (0.398–1.811%); (99.70–102.24%) and (0.475–1.474%) respectively, demonstrating a good accuracy of the used method.

**Table 2:** Evaluation of the accuracy and precision of the proposed method for determination of FVI, PAR and VC in human serum sample.

Studied compounds	Concentration added (µM)	Concentration found (µM)	Recovery (%)	Standard deviation × 10^−8^	Standard error × 10^−8^	Confidence level × 10^−8^	RSD (%)
FVI	0.2	0.204	101.79	0.131	0.075	0.324	1.609
	0.4	0.411	102.71	0.106	0.061	0.262	1.214
	0.8	0.818	102.27	0.110	0.063	0.273	1.110
	4	3.961	99.04	0.142	0.082	0.352	0.748
	10	9.942	99.42	0.263	0.152	0.651	0.724
PAR	0.5	0.505	101.06	0.025	0.014	0.061	1.755
	4	4.026	100.66	0.012	0.007	0.031	0.398
	8	8.089	101.12	0.084	0.049	0.209	1.670
	10	10.051	100.51	0.108	0.063	0.269	1.811
	15	14.858	99.05	0.134	0.077	0.333	1.619
VC	5	5.112	102.24	0.022	0.013	0.054	1.296
	10	10.043	100.43	0.015	0.009	0.038	0.697
	30	29.910	99.70	0.061	0.035	0.150	1.474
	40	39.885	99.71	0.059	0.034	0.147	1.163
	50	49.925	99.85	0.029	0.017	0.071	0.475

### Determination of FVI, PAR and VC in pharmaceutical samples

In this section we evaluate the proposed method for determination of pure FVI content in commercial tablets without interference from excipient materials. An oral tablet containing FVI (5 mg/tablet) is ground into fine powder and then dissolved in PBS/(pH 7.0) with a starting concentration of 0.5 mM. The FVI amount in the sample solution is determined by the standard additions. In the electrolytic cell we inject a standard with concentration 2.0 µM by micro-syringe. Then aliquots of the FVI tablet sample solution are prepared with concentrations ranging from (4 µM to 80 µM) and injected into the electrolytic cell. We use the following equation to calculate the concentration of the sample after each addition: [standard (2 µM)] + [tablet content solution added (4 µM to 80 µM)]. DPV mode is used to estimate the percentage of recovery values. The found concentrations are calculated from the average of three repeated measurements and are summarized in Table 3. The corresponding recovery values are in the range (98.20–103.35%), and the relative standard deviation values are in the range (0.119–2.48%) for the electrochemical quantitative determination of FVI in commercial tablet sample, indicating that the proposed method has a good accuracy. The same procedure was followed for determination of pure PAR and VC contents in their commercial tablets without interference from excipient materials. The data are listed in Table 3 with recovery values are in the ranges (99.98–101.7%), and (97.90–104.7%); and the relative standard deviation values are in the ranges (0.450–1.153%), and (1.123–2.929%), respectively.

**Table 3:** Evaluation of the accuracy and precision of the proposed method for determination of FVI, PAR and VC in drug formulations.

Drug	[Tablet] taken (µM)		[Standard] added (µM)		[Found] (µM)	Recovery (%)	RSD (%)
FVI	4		2		6.201	103.35	1.61
	8		2		10.166	101.66	2.48
	20		2		22.704	103.20	1.583
	30		2		31.619	98.81	1.869
	80		2		80.522	98.20	0.119
PAR	20		2		22.062	100.28	0.450
	25		2		26.994	99.98	1.090
	30		2		32.551	101.72	1.153
	20		2		22.062	100.28	0.450
	25		2		26.994	99.98	1.090
VC	20		2		23.043	104.74	2.929
	30		2		33.015	103.17	1.523
	40		2		41.961	99.91	1.590
	50		2		50.908	97.90	1.341
	60		2		61.905	99.85	1.123

## Conclusion

A novel electrochemical method is applied to determine FVI, PAR and VC in human serum samples. The sensor is constructed by casting a thin layer of CNTs followed by electrodeposition of another layer of β-CD over a GC electrode surface. The combined effect of large conducting surface area of CNTs and host–guest interaction ability of β-CD with FVI improves the sensing performance of the sensor. β-CD molecules increase the composite surface area and the contact areas with the FVI. Further, β-CD accumulates FVI due to the hydrophobicity of the inner core, and the hydrophilic outer core facilitates more oxidation of FVI. The β-CD structure contains relatively large number of oxygen atoms and OH groups. Hydrogen bond (weak non-covalent interaction) is formed between the OH group of β-CD and the OH group of FVI. The hydrogen bond activates and decreases the bonding energy of the hydroxyl group of FVI and facilitates the electron transfer reaction, which results in enhancing the rate of electron transfer process. The sensor in human serum shows excellent catalytic effect for simultaneous determination of FVI, PAR and VC in human serum sample. Also, the GC/CNT/CD electrode is effective in separating and detecting the FVI in presence of interfering compounds such as AA, and UA. Additionally, the sensor is capable of reliably detecting FVI in clinical trial samples and pharmaceutical formulations. The limitation of this method is that the sensor cannot detect simultaneously in the drugs mixture solution other species having similar oxidation potentials.

### Supplementary Information

Supplementary Information.

## Data Availability

All data generated or analyzed during this study are included in this published article [and its supplementary information files].
