# Supplementary material for: Electrochemical sensor for simultaneous determination of antiviral favipiravir drug, paracetamol and vitamin C based on host–guest inclusion complex of β-CD/CNTs nanocomposite
Source: Sci Rep. 2023 Nov 14;13:19910. doi: 10.1038/s41598-023-45353-3 (PMC10645768; doi:10.1038/s41598-023-45353-3)
Supplement: Supplementary file 1 — Supplementary Information. [file 41598_2023_45353_MOESM1_ESM.docx]

**Supplement Table 1:** The devices used in this work.

| **Instrument** | **Uses** |
| --- | --- |
| BAS-Epsilon electrochemical equipment with a cell contained three electrodes (GCE (Ф: 3 mm), platinum wire and Ag/AgCl (3 M KCl). | Voltammetry measurements |
| Quanta 250 FEG instrument | Scanning electron microscopy FE-SEM measurement |
| Quanta 250 FEG instrument | Energy dispersive X-ray spectroscopy (EDAX) measurement |
| Fourier transforms infrared (FTIR) Shimadzu IR-Affinity 1 spectrometer | Surface functional groups characterization |
| Atomic force microscope (Veeco) | Surface area measurements and morphology |

**Supplement Table 2:** Comparison of the analytical parameters for FVI determination using GC/CNT/CD electrode with previously cited electrodes.

| Modified electrodes | pH | Linearity range  (µM) | Detection limit  (µM) | Sensitivity  (μA/μM) |
| --- | --- | --- | --- | --- |
| CPT-BDDE^21^ | 8.0 | 0.64 – 130 | 1.5 × 10^-3^ | 0.00231 |
| NIP-Co/Ni@MOF/SPE^54^ | 4.0 | 0.01 – 14.64 | 8.17 × 10^-3^ | 0.187 |
| PGE^58^ | 5.0 | 5.0 – 200 | 1.55 | 0.626 |
| MnO_2_-rGO/SPE^59^ | 3.0 | 0.01 – 55 | 9.1 × 10^-3^ | 0.048 |
| Diamond NPs/CPE^60^ | 4.0 | 0.2 – 1.0 | 4.83 × 10^-3^ | 0.0126 |
| GCE^61^ | 10 | 6.4 – 640 | 1.7 | 0.0799 |
| GC/CNT/CD | 7.0 | 0.07 – 100 | 1.09 × 10^-3^ | 0.197 |

PGE: pencil graphite electrode; NIP-Co/Ni@MOF/SPE: Molecularly Imprinted Polymer Based on the Bimetallic Metal-Organic Framework; CPT-BDDE: cationic surfactant media using a boron-doped diamond electrode; MnO_2_-rGO/SPE: Molecularly Imprinted Polymer Based on manganese oxide and graphene; Diamond NPs/CPE: Diamond Nanoparticles-based carbon paste electrode.


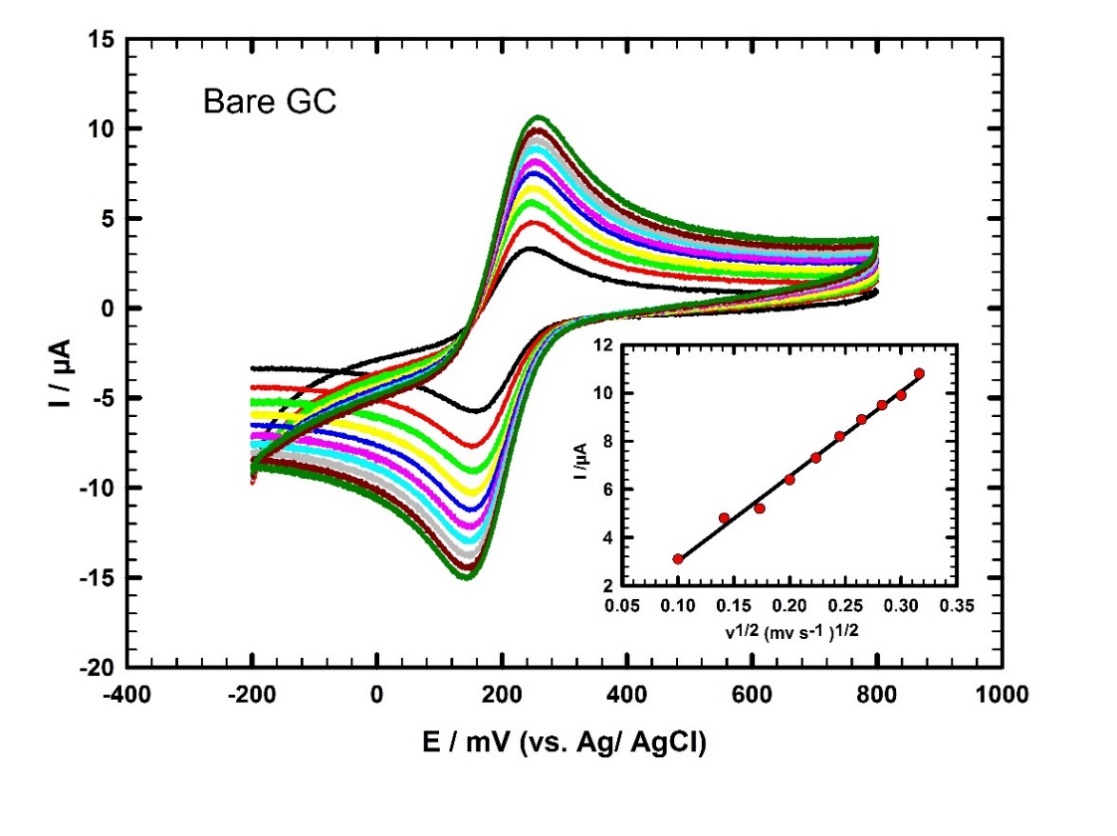


**(A)**


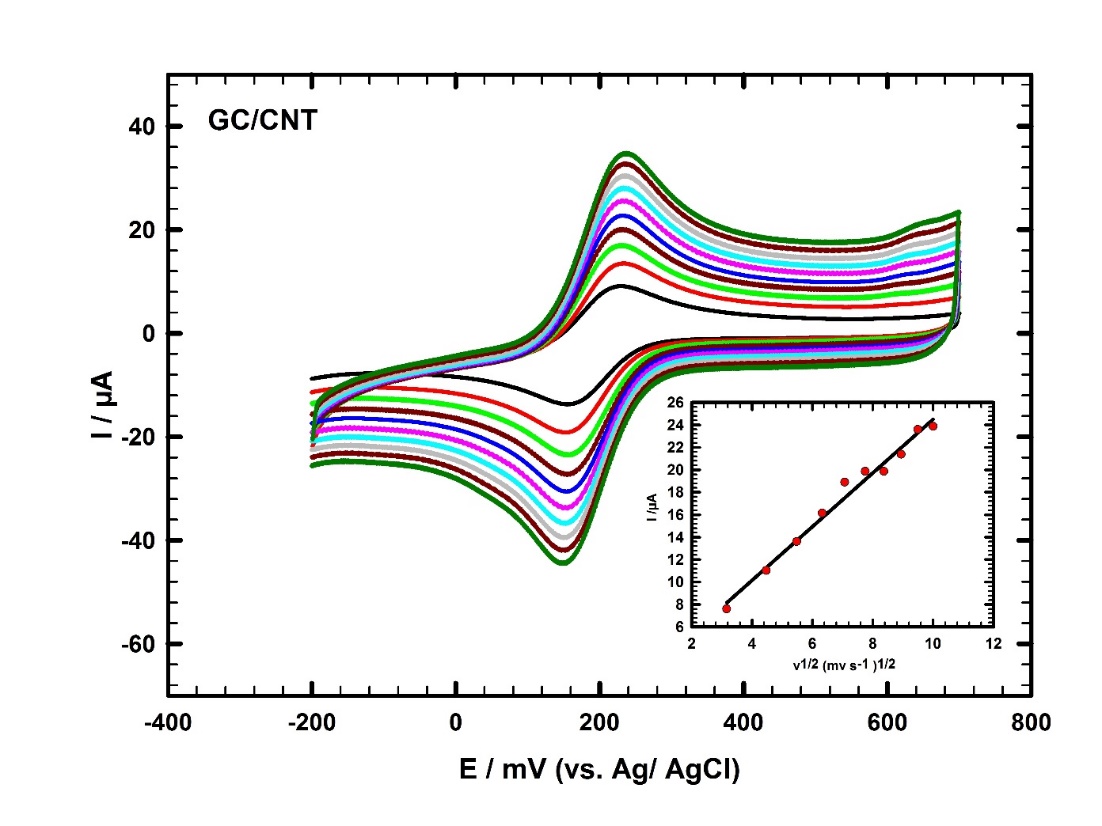


**(B)**


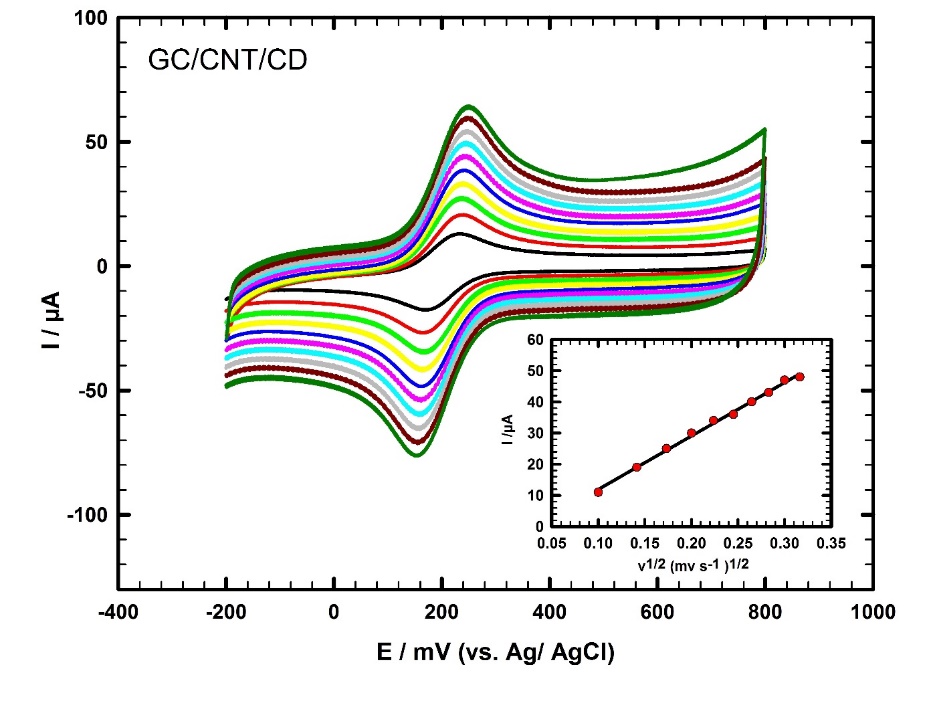


**(C)**

**Supplement Figure 1 (A-C):** CV experiments in 1.0 mM K_3_[Fe(CN)_6_] system using different electrodes GC,GC/CNT, GC/CNT/CD, respectively. Insets: Corresponding [relations between oxidation peak current and square root of scan rate](#_Toc136482542).


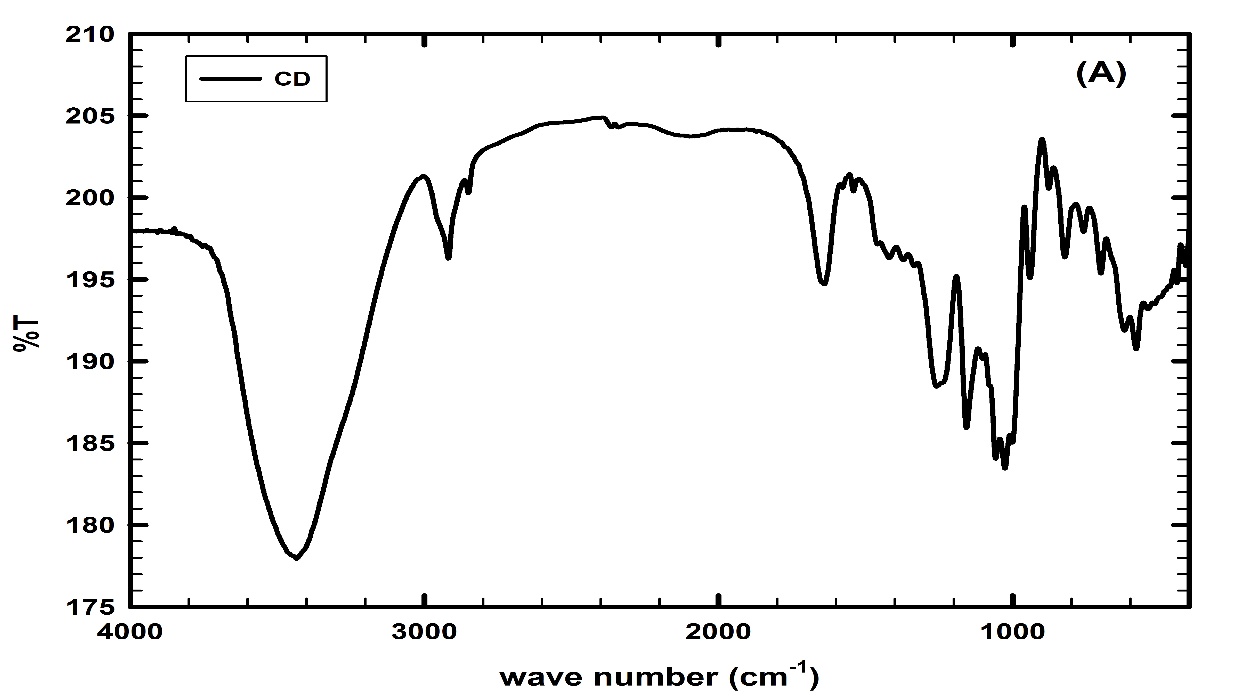


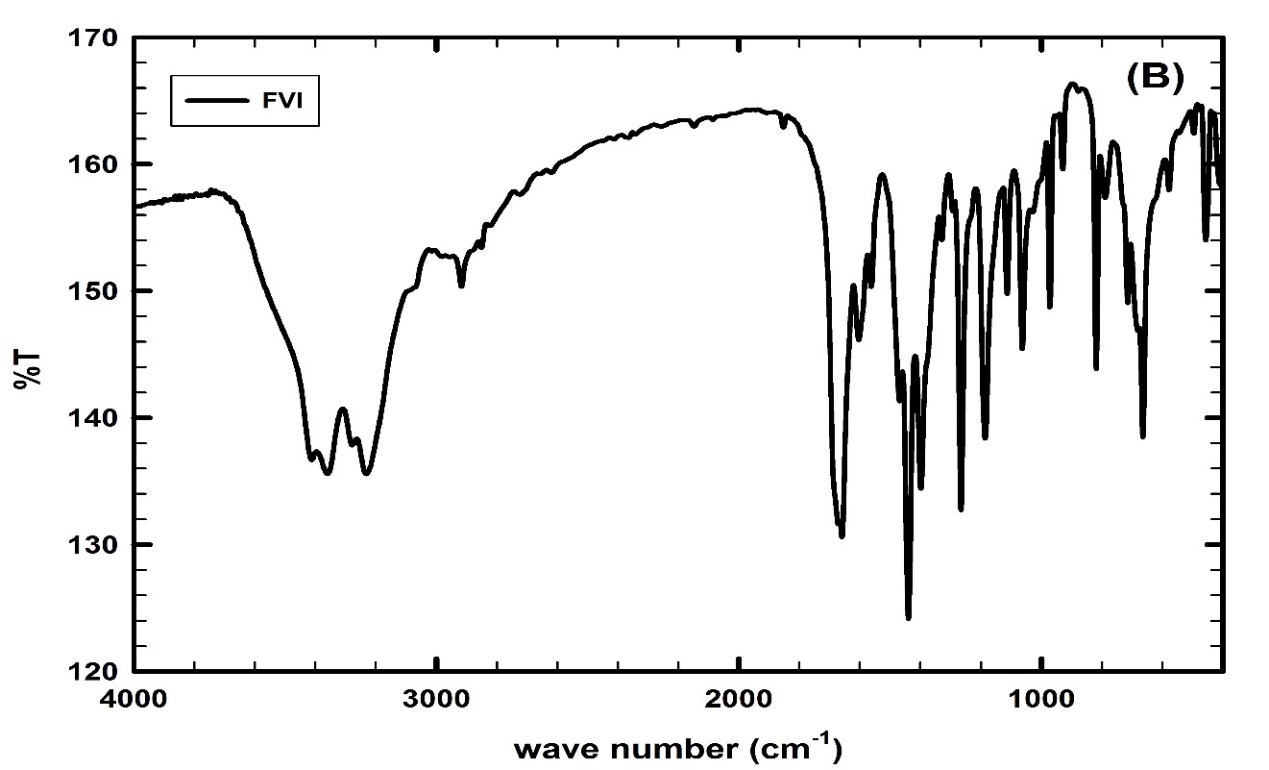


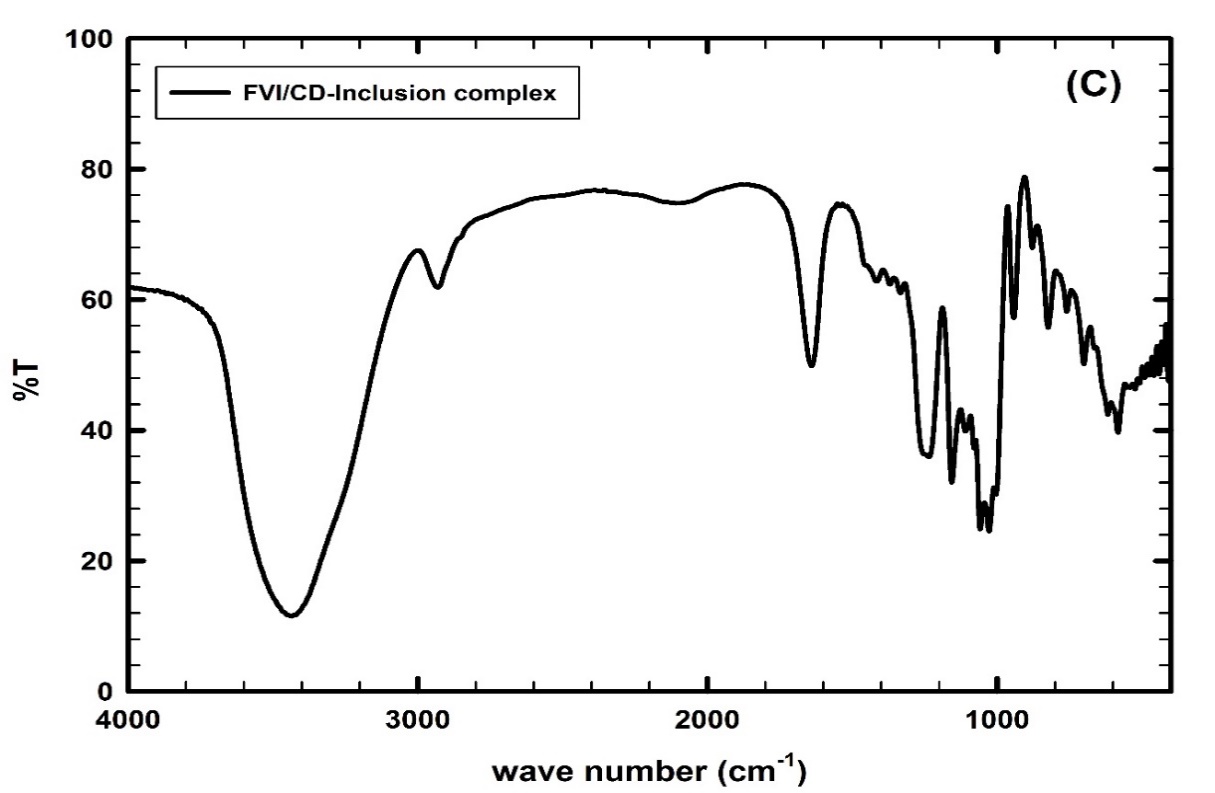


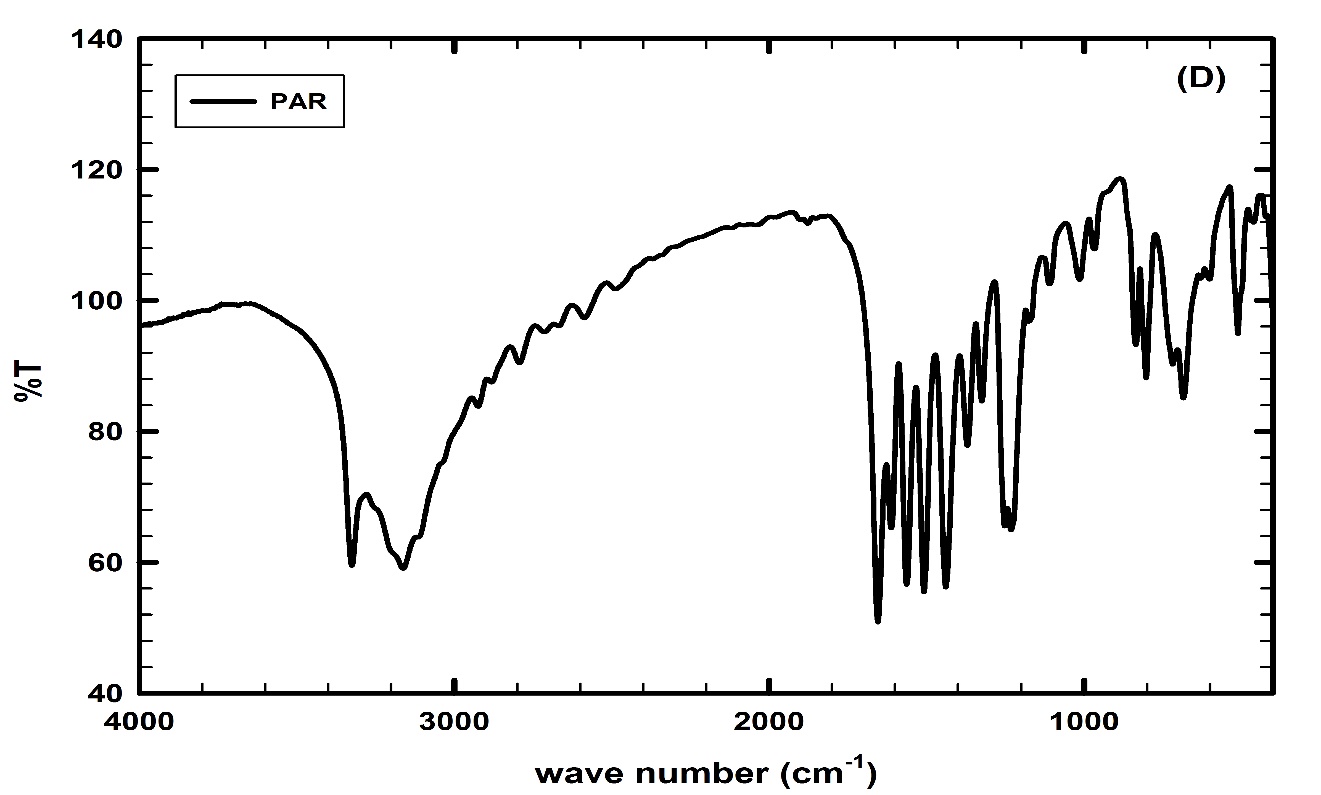


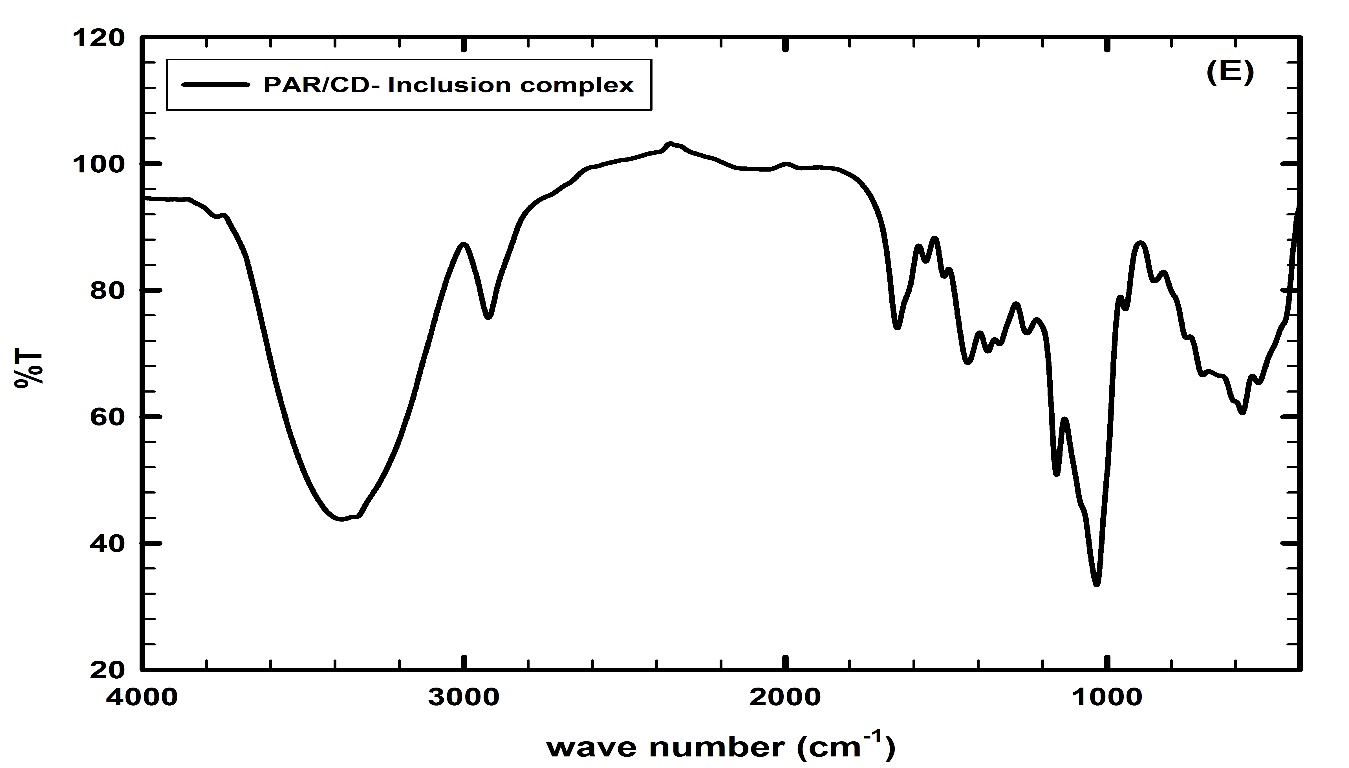


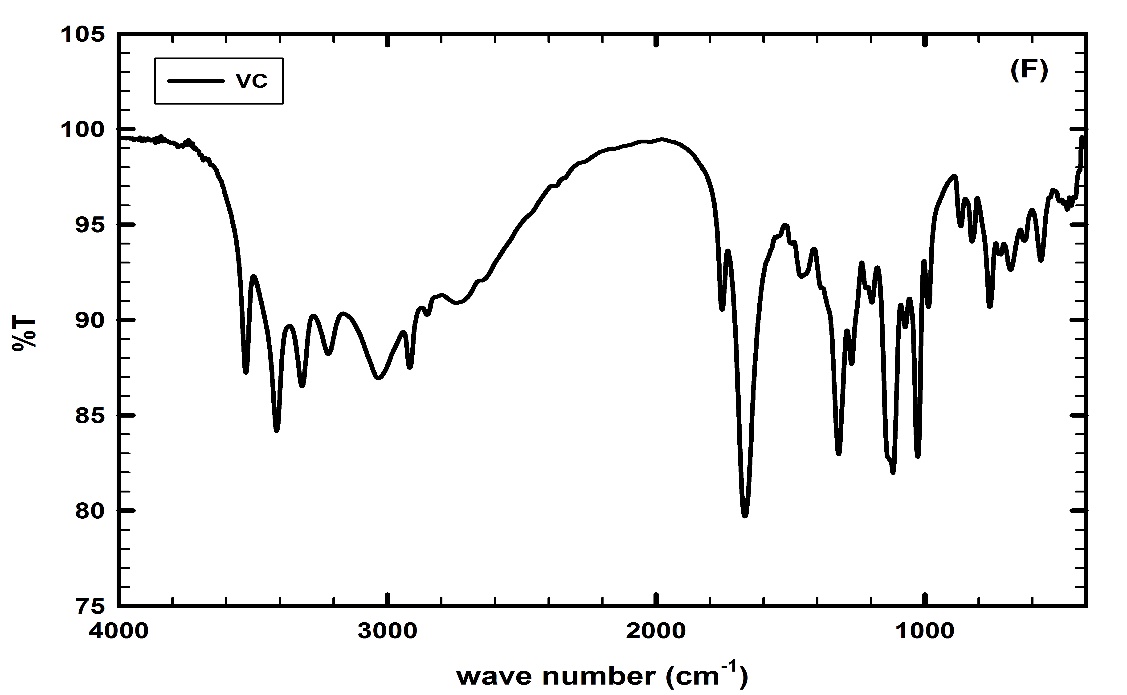


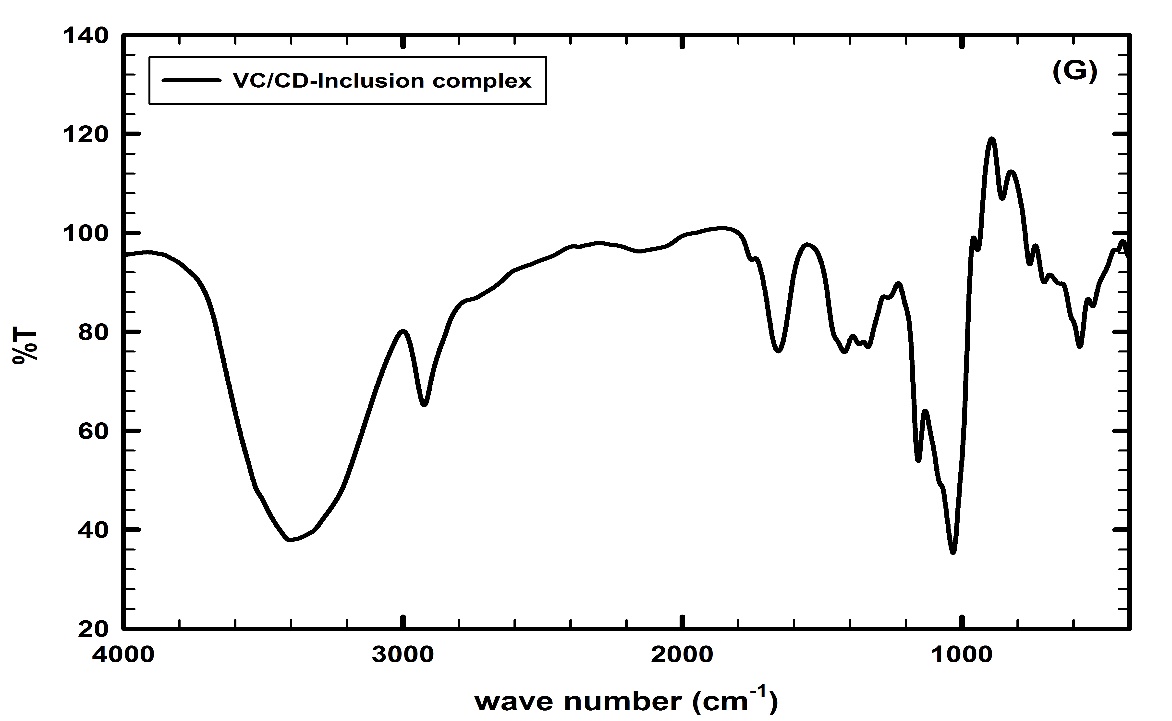


**Supplement Figure 2** **(A-G):** FTIR of β-CD, FVI, inclusion complex of FVI; PAR, inclusion complex of PAR; VC, and inclusion complex of VC, respectively.


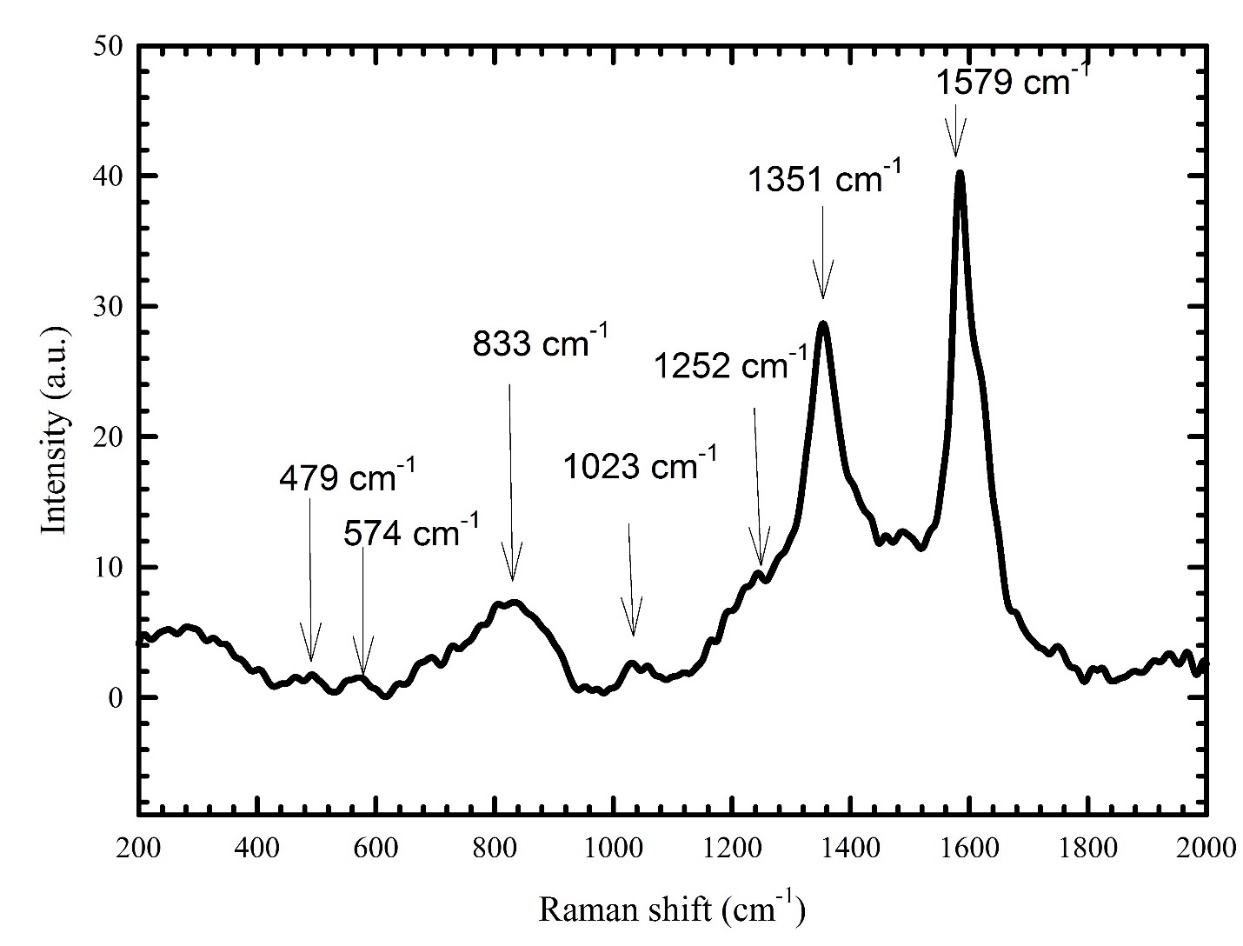


**Supplement Figure 2 H:** Raman spectrum of CNT/CD composite.

Analyzing the spectrum of CNT/CD composite, β-CD bands appear around 1252 cm^-1^, 1023 cm^-1^, 833 cm^-1^, 574 cm^-1^, and 479 cm^-1^ are related to C-H stretch, alcohol hydroxyls, C-O-C bonds between glucoses. CNTs are recognized by D and G bands appear at 1351 cm^-1^ and 1579 cm^-1^, respectively.


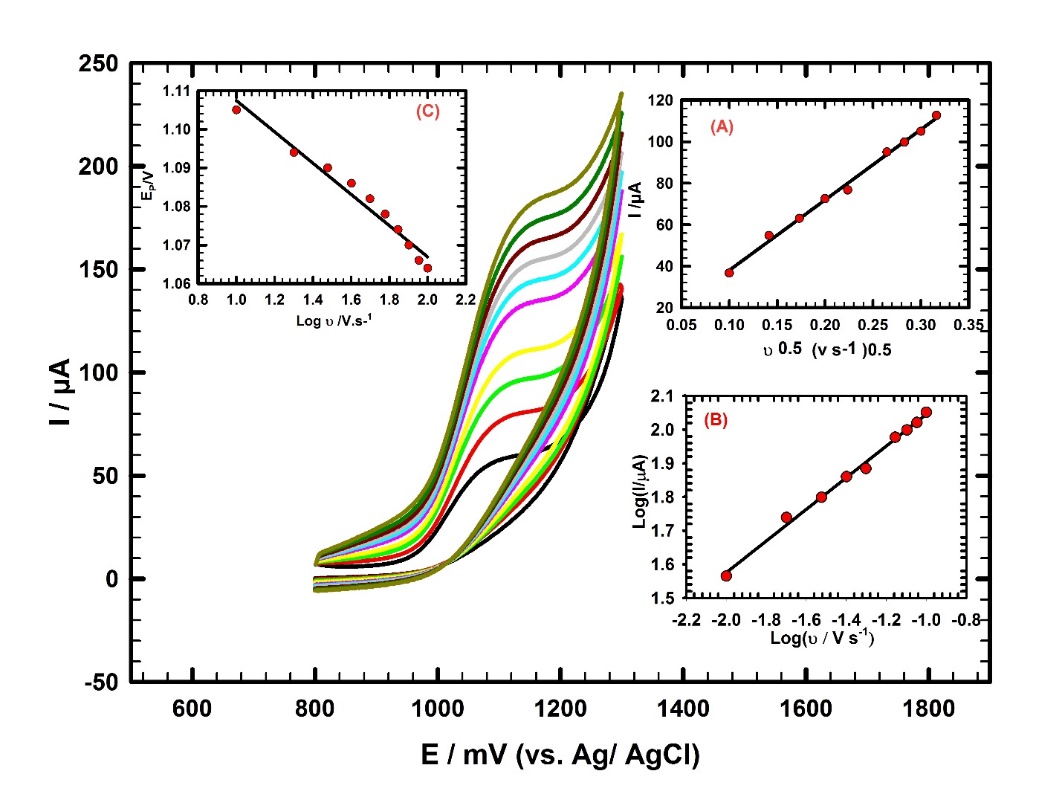


**Supplement Figure** **3:** Effect of varying the scan rates on the CVs of 1.0 mM FVI in 0.1 M PBS (pH 7.0). Insets: **(A)** Relation between oxidation peak current and square root of scan rate. **(B)** Relation between log oxidation peak current and log scan rate. **(C)** Relation between oxidation peak potential and log scan rate.


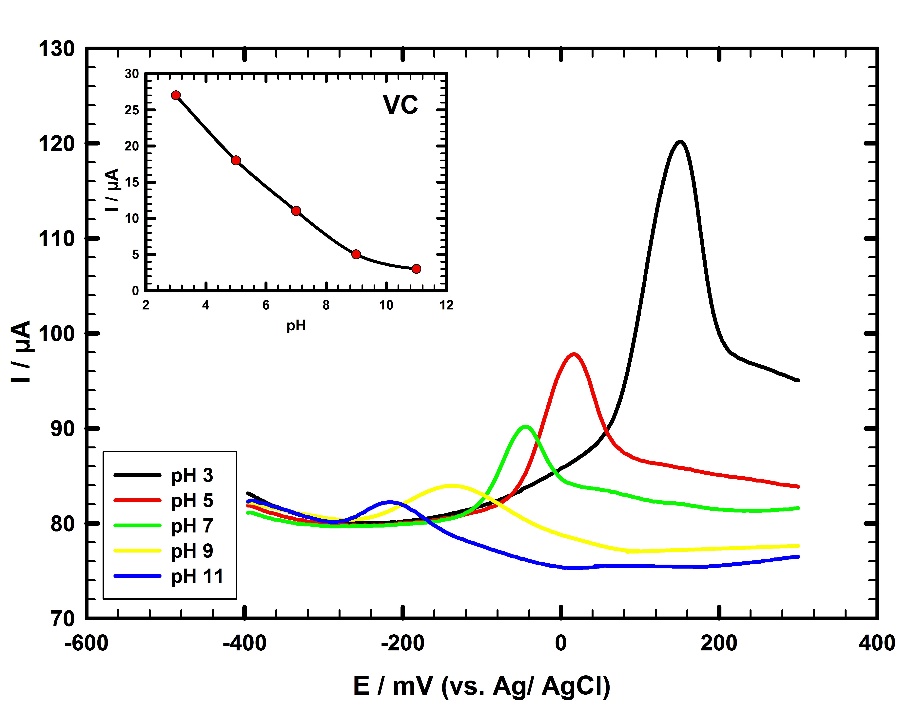


**(A)**


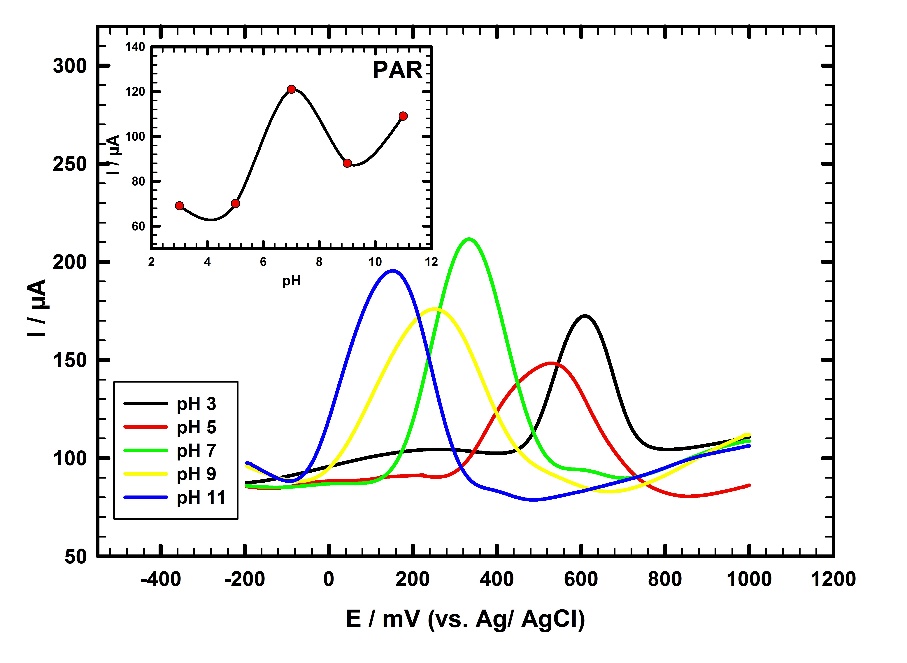


**(B)**

**Supplement Figure 4: (A):** DPVs of 1.0 mM VC/0.1 M PBS with different pH values using GC/CNT/CD electrode. **(B):** DPVs of 1.0 mM PAR/0.1 M PBS with different pH values using GC/CNT/CD electrode. Insets: Corresponding relations between peak current and pH.


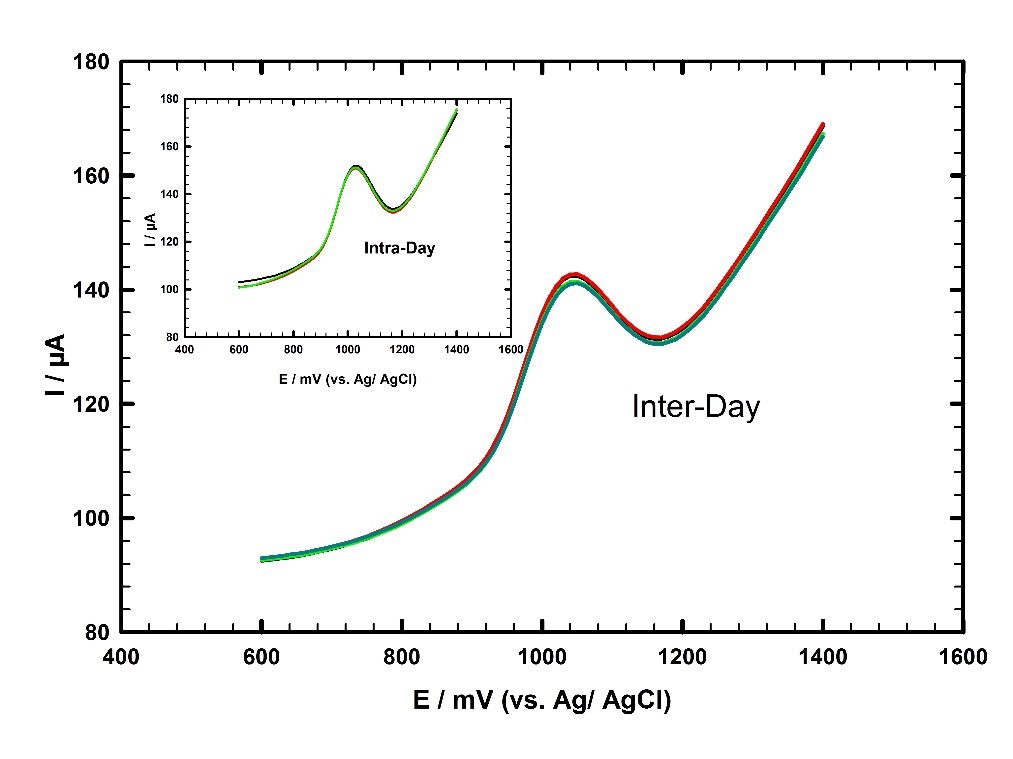


**(A)**


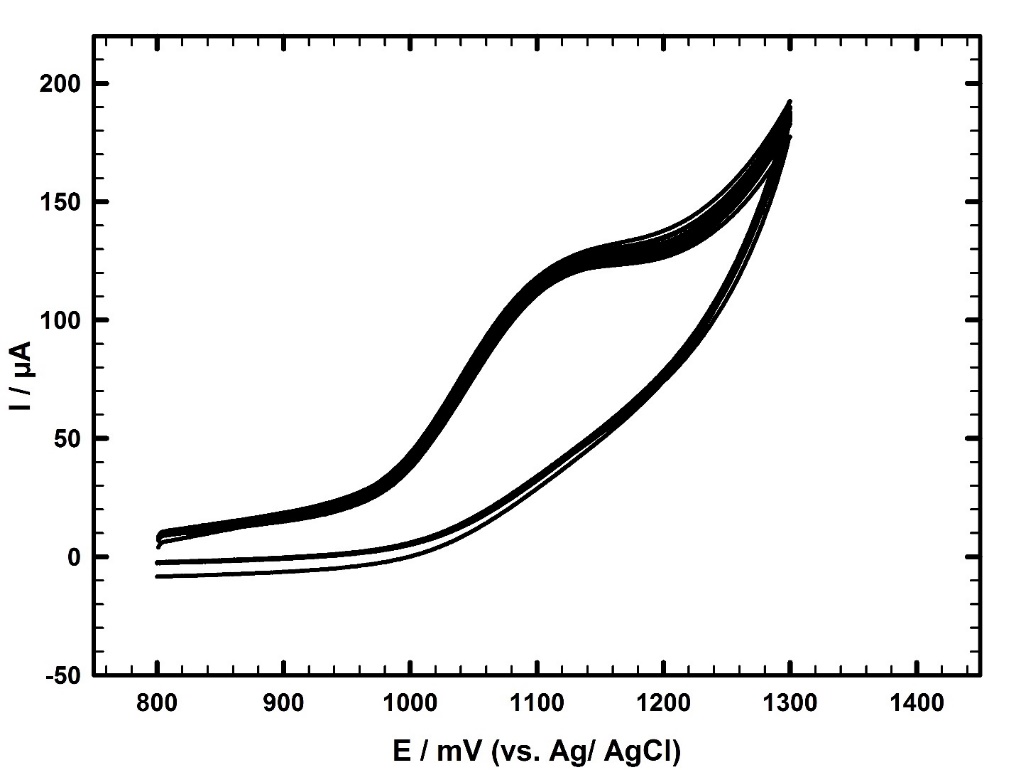


**(B)**


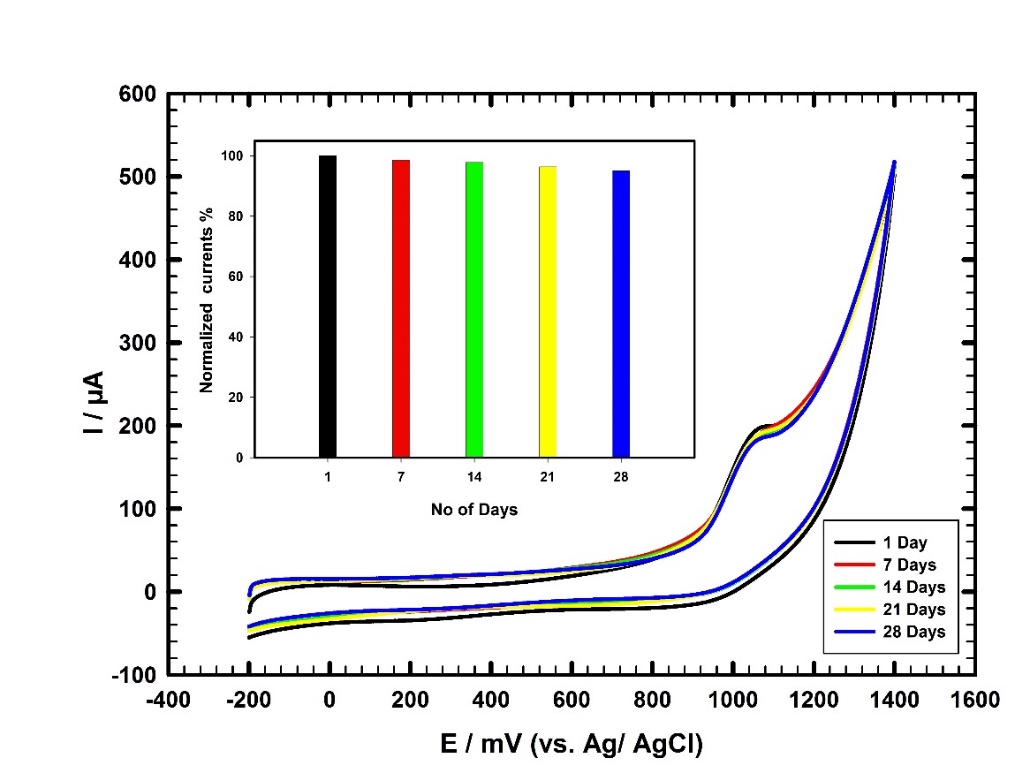


**(C)**


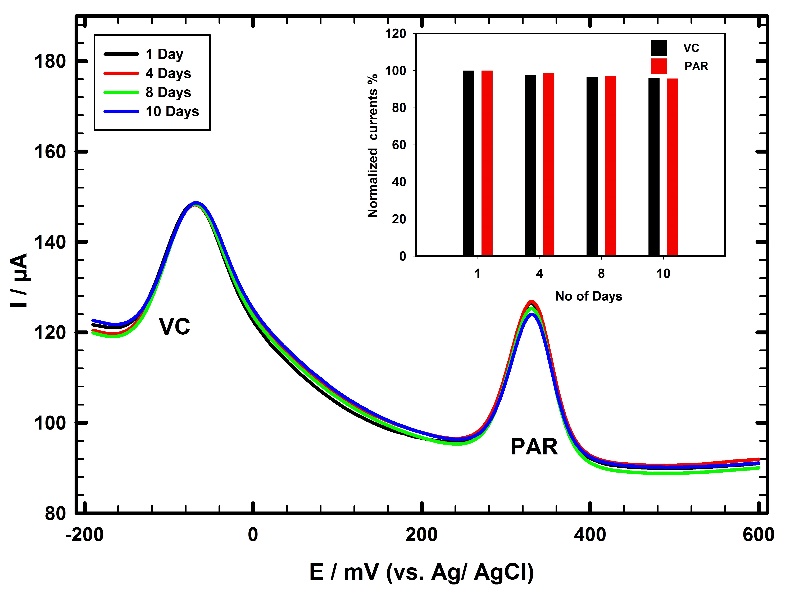


**(D)**

**Supplement Figure 5A):** The inter-day reproducibility of GC/CNT/CD electrode in 0.1 mM FVI/0.1 M PBS (pH 7.0). Inset: The intra-day reproducibility of GC/CNT/CD electrode in 0.1 mM FVI/0.1 M PBS (pH 7.0). **B):** Repeated CVs (25 cycles) for testing the stability using GC/CNT/CD electrode in 1.0 mM FVI/0.1 M PBS (pH 7.0); scan rate 50 is mV.s^–1^. **C):** Electrochemical response of the electrode for long term stability (28 days) using GC/CNT/CD electrode in 1.0 mM FVI/0.1 M PBS (pH 7.0); scan rate 50 mV.s^–1^. **D):** Electrochemical response of the electrode for long term stability (10 days) using GC/CNT/CD electrode in 1.0 mM VC and 0.1 mM PAR/0.1 M PBS (pH 7.0).


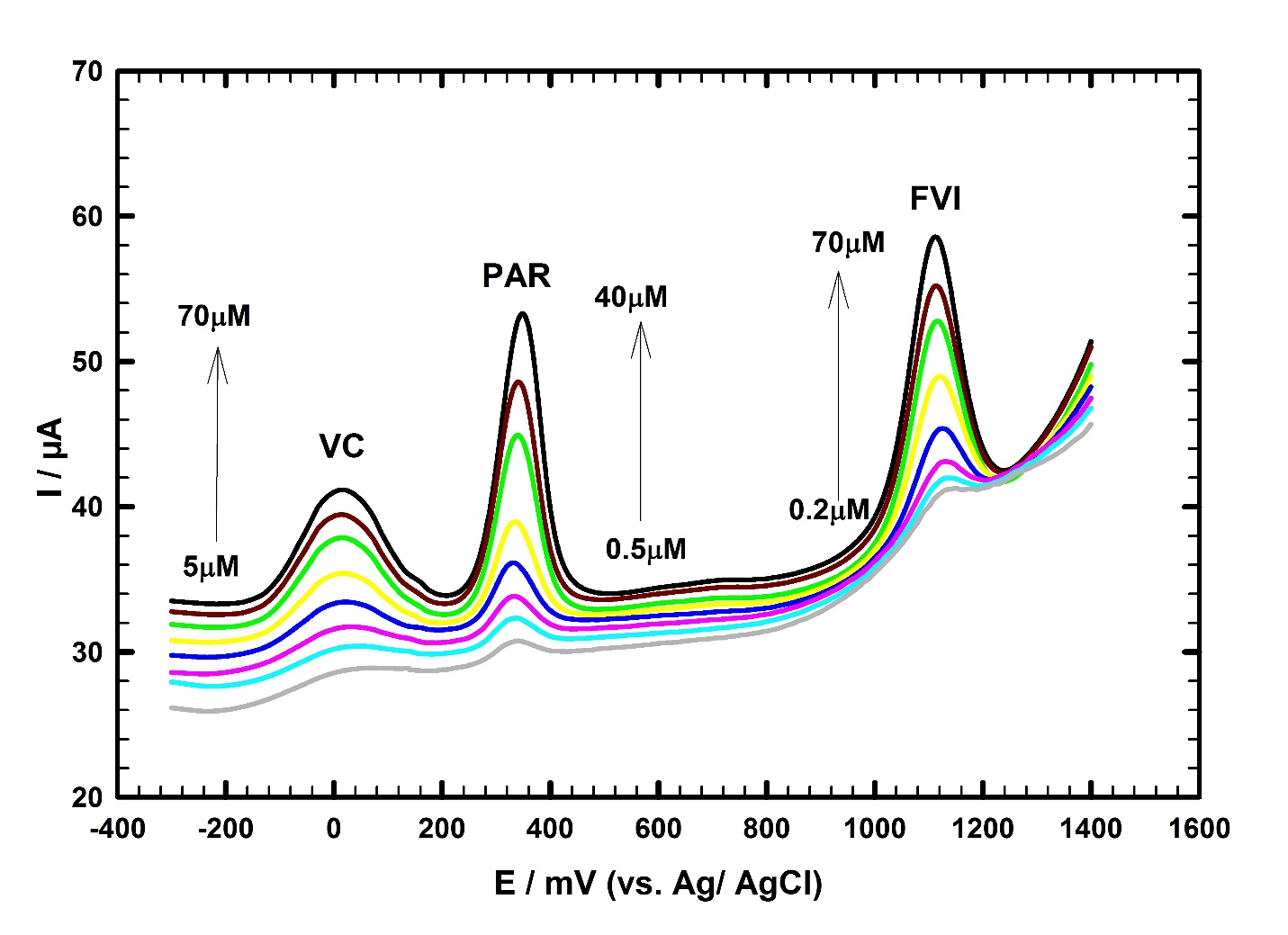


**Supplement Figure 6:** DPVs of simultaneous determination of FVI, PAR and VC in the concentration ranges (0.2→ 70 µM), (0.5→40 µM), and (5→70 µM) in dilute human serum/0.1 M PBS pH 7.0.
